# Abrupt cooling over the North Atlantic in modern climate models

**DOI:** 10.1038/ncomms14375

**Published:** 2017-02-15

**Authors:** Giovanni Sgubin, Didier Swingedouw, Sybren Drijfhout, Yannick Mary, Amine Bennabi

**Affiliations:** 1 Laboratoire des Sciences du Climat et de l'Environnement (LSCE), Institut Pierre Simon Laplace (IPSL), 91191 Gif-sur-Yvette, France; 2 Environnements et Paleoenvironnements Oceaniques et Continenteaux (EPOC), UMR CNRS 5805, Université de Bordeaux, 33615 Pessac, France; 3 Royal Netherlands Meteorological Institute (KNMI), 3730AE De Bilt, The Netherlands; 4 National Oceanography Centre (NOC), University of Southampton, Southampton SO14 3ZH, UK; 5 Institut de Mecanique et d'Ingenierie (I2M), Université de Bordeaux, 33615 Pessac, France

## Abstract

Observations over the 20th century evidence no long-term warming in the subpolar North Atlantic (SPG). This region even experienced a rapid cooling around 1970, raising a debate over its potential reoccurrence. Here we assess the risk of future abrupt SPG cooling in 40 climate models from the fifth Coupled Model Intercomparison Project (CMIP5). Contrary to the long-term SPG warming trend evidenced by most of the models, 17.5% of the models (7/40) project a rapid SPG cooling, consistent with a collapse of the local deep-ocean convection. Uncertainty in projections is associated with the models’ varying capability in simulating the present-day SPG stratification, whose realistic reproduction appears a necessary condition for the onset of a convection collapse. This event occurs in 45.5% of the 11 models best able to simulate the observed SPG stratification. Thus, due to systematic model biases, the CMIP5 ensemble as a whole underestimates the chance of future abrupt SPG cooling, entailing crucial implications for observation and adaptation policy.

## Abstract (web summary)

Concerns on climate change include the risk of abrupt cooling in the North Atlantic. Here, the authors analyse CMIP5 projections and show that a convection collapse in the subpolar gyre can cool this region by up to 3°C in 10 years, which is as likely to occur by 2100 as a continuous warming.

The increase of the concentration of greenhouse gases in the atmosphere since the industrial era^1^ has led to an Earth radiative imbalance and an accumulation of energy within the climate system^2^. Most of this energy has been absorbed by the ocean^3^,^4^ through heat uptake, contributing to the gradual rise of the Sea Surface Temperature (SST) observed in the 20th century^5^. Moreover, warmer conditions have enhanced the hydrological cycle^6^, making the net evaporative subtropical regions saltier and the subpolar regions fresher^7^. The extent and time-scale of the oceanic response over the last century has varied regionally, with an amplified SST increase in the Arctic, the Nordic Seas and the western boundary current regions^8^, and a subdued warming trend over the North Atlantic (NA) subpolar gyre (SPG)^9^. The latter, including the Labrador and Irminger Seas, showed a cooling trend over the last century in spite of global warming^10^,^11^.

The NA is a site of deep convection and dense water formation feeding the Atlantic meridional overturning circulation (AMOC), which is responsible for most of the northward heat transport in the Atlantic Ocean^12^. A classical diagnostic for the convective activity is the mixed layer depth (MLD, see Methods). Its observation-based pattern from GLORYS Reanalysis data^13^, shown in Fig. 1, highlights the SPG and the Nordic Seas to be the main regions of deep-water formation in the NA. Sinking of surface water is essential in sustaining the AMOC by connecting warm and saline northward surface currents with the returning cold deep currents. While sinking mainly occurs in the boundary current encircling the convection site^14^, deep convection and net sinking appear tightly coupled, witnessed by model experiments in which the AMOC collapses when oceanic deep convection stops due to freshwater hosing^15^. Deep water in the SPG forms during the winter when oceanic heat loss decreases the density stratification^16^, thus destabilizing the water column. Convective processes increase the thickness of the MLD, and link the surface water masses to the deep ocean. The MLD, in turn, is closely connected to the SPG cyclonic circulation, which is driven by both cyclonic wind shear and buoyancy forcing. This cyclonic circulation around the dense core of the gyre modulates the inflow of salty water from the subtropics^17^. Moreover, it promotes isopycnal outcropping at the centre of the SPG^16^, thus exposing weakly stratified water masses underneath the surface to atmospheric conditions. This upward doming of isopycnals represents a preconditioning for deep convection. As a result, an intense SPG circulation favours a deep mixed layer and strong convection, allowing heat exchanges between the deep ocean and the atmosphere. By contrast, a weaker SPG circulation limits the active mixed layer, thus confining the oceanic heat loss to shallower depths and yielding more modest SPG deep-water formation. Because the SPG responds to changes in the MLD^18^, a positive feedback between the two can arise, leading to potential instabilities in the SPG system.

The 20th century warming and freshening of the NA may have weakened the AMOC^19^,^20^,^21^, raising concerns about its stability^22^,^23^,^24^ and the risk of future disruptions^25^,^26^. If the AMOC imports salt into the Atlantic, its slowdown could trigger a positive feedback by amplifying the freshening over the convection sites^22^,^27^,^28^. This feature makes the AMOC one of the potential tipping elements of the climate system^29^, which can experience a drastic shift in response to global warming when a certain climatic threshold is passed^24^. An AMOC disruption is conditional on the interruption of deep-water formation over both the main convective sites, and would cause a cooling over the whole NA due to the collapsed northward heat transport^25^,^26^ normally accomplished by the overturning circulation. However, an AMOC shutdown has been estimated as ‘very unlikely to occur in the 21st century’ in the latest IPCC AR5 report^1^,^30^.

In parallel, both conceptual and coupled climate models have highlighted the potential bistability of the SPG circulation, which may switch from a strong mode to a weak mode^31^,^32^ due to positive feedback mechanisms involving the stratification at the centre of the gyre. The ongoing SPG freshening^33^ may increase the background stratification^34^,^35^ in this region, weakening the local deep convection^36^. This, in turn, has the potential to push the SPG cyclonic circulation towards a persistent weak state due to a reduced buoyancy forcing. At the same time, a weaker cyclonic circulation amplifies the stratification within the SPG, because of a decreased inflow of saltier water from the surrounding regions. The interplay of these feedback mechanisms may further inhibit the local deep convection up to its permanent collapse, thus provoking a local abrupt cooling due to a drastically reduced MLD^37^ and the associated reduction in heat transfer from the deep ocean to the surface. Between 1968 and 1972 the SPG experienced such a rapid drop in SST^38^, which was linked to the large freshwater anomaly observed in the region^39^, known as the Great Salinity Anomaly^40^. The latter coincided with an unusually shallow mixed layer, limiting oceanic heat loss^41^ and raising concerns about the stability of the SPG convective activity and its potential collapse under global warming conditions^31^,^32^,^37^. However, contrary to a potential AMOC disruption, no assessment has been made of the possibility of a local SPG convection collapse in the latest IPCC AR5 report.

Here we assess such a possibility in 40 state-of-the-art coupled climate models, also accounting for the impact of model bias on future projections through a comparison with present-day observations. We find that the increase in radiative forcing may provoke abrupt non-linear shifts in SPG dynamics, the latter being characterized by a permanent collapse of the local convective. This occurs in various climate change projections for a few climate models showing a good ability to represent the present-day stratification of the subpolar NA. The collapse of convective activity leads to an abrupt cooling of a few degrees within 10 years, directly impacting North American and European climate, raising concerns about climate change adaptation policies in these areas.

## Results

### Different SST responses in the SPG

**Figure 1:**
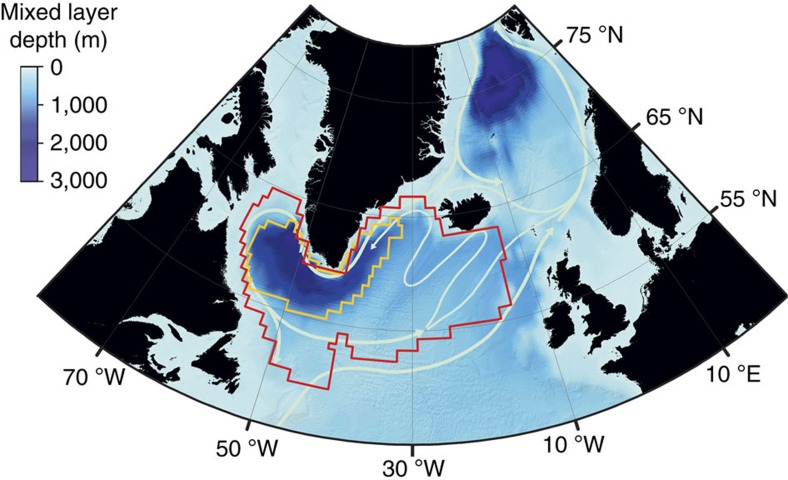
Sites of deep-water formation in the North Atlantic. Map of the maximum winter mixed layer depth (m) averaged over the 1993–2012 period according to the GLORYS reanalysis. The red contour represents the reference area for our analysis (see Method*s)*. Its total surface measures 3.61 × 10^6^ km^2^ and it entirely spans the subpolar NA, including those sites in the Labrador and Irminger Seas that are regularly subject to convective activity. The yellow contour highlights the region for which the maximum MLD averaged over the 1993–2012 period exceeds 1,000 m. This area has been used for a sensitivity test of our main findings on the particular choice of the reference region (Supplementary Fig. 9). Arrows indicate the main surface currents, including the North Atlantic Current, the western subpolar gyre in the Labrador and Irminger Seas and the eastern subpolar gyre in the Nordic Seas.

**Figure 2:**
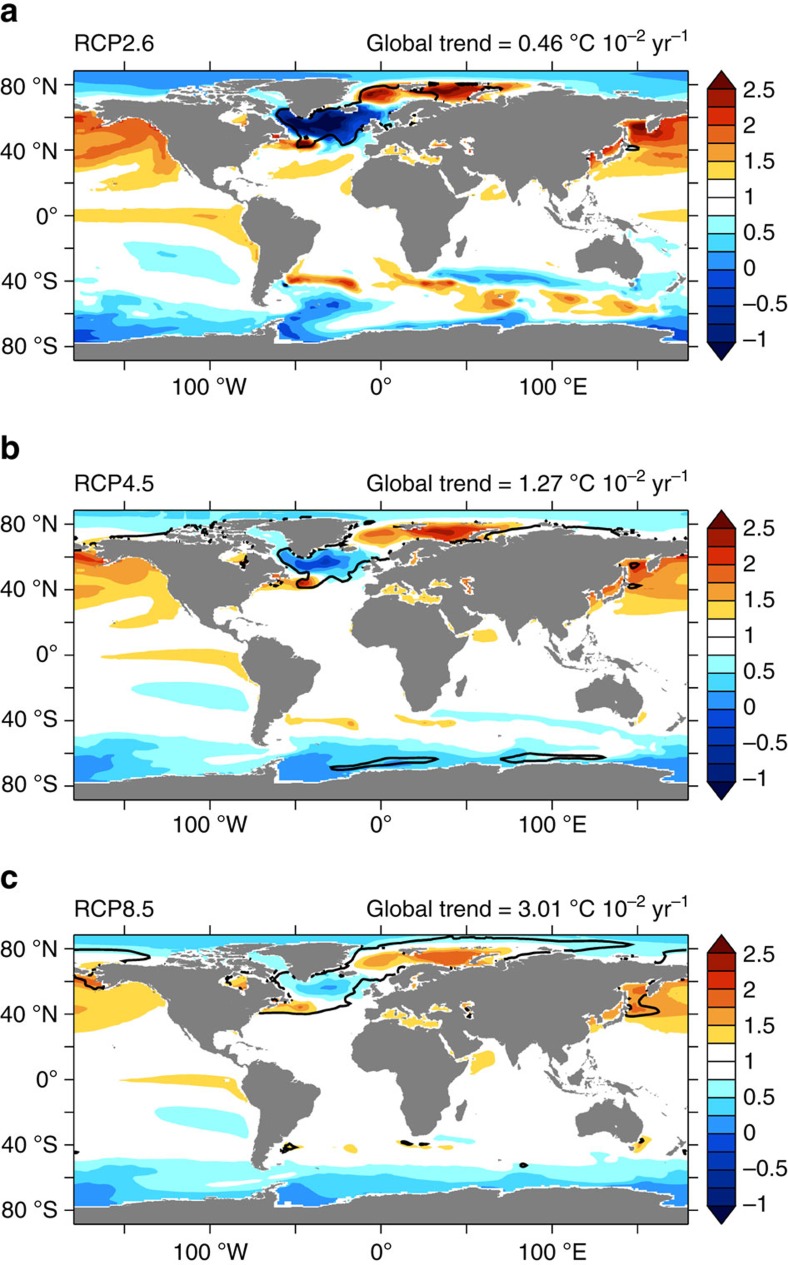
Patterns of SST response in RCP scenarios. Ensemble mean of the 21st century SST trend normalized by its own global mean (dimensionless quantity) for (**a**) RCP2.6 simulations, (**b**) RCP4.5 simulations and (**c**) RCP8.5 simulations. The globally averaged SST trend ensemble mean is indicated for each scenario, that is, 0.46 10^−2^ ^o^C year^−1^ for the RCP2.6 experiments, 1.27 10^−2^ ^o^C year^−1^ for the RCP4.5 experiments and 3.01 10^−2^ ^o^C year^−1^ for the RCP8.5 experiments. Since the globally averaged SST trend ensemble mean is positive for all scenarios, the non-dimensional value in each grid point is >1 when characterized by amplified warming, <1 when characterized by a subdued warming and <0 when characterized by cooling. The black contour shows regions with maximum ensemble spread (see Methods).

We investigated projections from 40 climate models participating in the fifth Coupled Model Intercomparison Project (CMIP5) under different emission scenarios (RCPs)^42^. We systematically scanned 27 RCP2.6 simulations, 39 RCP4.5 simulations and 40 RCP8.5 simulations for a total of 106 experiments. In each case, the analysis also included the preceding historical simulation capturing all known radiative changes since 1860. For all projections a rise in the ensemble mean global SST was found (Fig. 2). The global SST trends are, respectively, 0.46±0.30 °C per century for the RCP2.6 ensemble, 1.27±0.39 °C per century for the RCP4.5 ensemble and 3.01±0.58 °C per century for the RCP8.5, thus continuing the warming trend observed over the last decades^43^,^44^. By normalizing the ensemble mean SST trend by the globally averaged value, the resulting pattern clearly reveals that the warming signal is not uniform in space (Fig. 2). Some regions experience an amplified SST increase, for example, the Nordic Seas, while other regions are characterized by a subdued warming trend, for example, the SPG. Moreover, the uncertainty in SST projections peaks over the NA convection regions (black contour in Fig. 2). In the SPG, 70% of the experiments feature an increase in SST, while the remaining 30% show a reversed trend (Supplementary Table 1). The area of the NA that for each RCP scenario is characterized by a subdued SST warming and a significant model uncertainty (Methods) roughly matches the SPG (red contour in Fig. 1). Our analysis focuses on this region to identify possible abrupt cooling events and to evaluate whether the models exhibiting such abrupt cooling are reliable.

### Abrupt cooling events in the SPG

We define here as ‘abrupt’ those cooling events in the SPG for which the 10-year SST decrease is at least three times larger than the standard deviation of its annual data in the pre-industrial simulation (Methods). We detected a total of 15 cases (14% of the available projections) satisfying our definition (Supplementary Fig. 1), involving nine different models (22.5% of the models). We identified two main processes driving an abrupt SPG cooling. In seven models (17.5% of the total) a rapid SST decrease in the SPG is driven by a sudden local MLD contraction, that is, a convection collapse, affecting but not completely disrupting the AMOC. In two models (5% of the total) the temperature drop involves the entire northern NA and is caused by a massive AMOC reduction and its associated change in meridional heat transport. Thus, although deep convection and AMOC are strictly connected, abrupt shifts in SPG convection may not necessarily imply similar AMOC shifts. Because deep convection in the Greenland-Iceland-Norwegian Sea and in the Labrador-Irminger Sea, as well as the overflows from Denmark Strait and the Scotland-Faroe channel are all integral parts of the AMOC deep-water formation system, a collapse in one part of this system may occur without an equally abrupt response in the AMOC. This supports the distinction between two separated climatic tipping points for the NA abrupt cooling, namely one associated with a local SPG convection collapse and one associated with a large-scale AMOC disruption.

### Three different types of SST response in the North Atlantic

Based on their different SPG projections, we discerned three different subsets of models (Table 1), namely those not showing any kind of abrupt cooling (the ‘non-abrupt’ ensemble, 31 models), those characterized by a convection collapse in the SPG (the ‘SPG convection collapse’ ensemble, seven models) and those simulating a collapse of the AMOC before year 2100 (the ‘AMOC disruption’ ensemble, two models). For the models projecting a rapid SST cooling over the SPG, the corresponding level of global warming and the year in which the abrupt change starts are also displayed (Table 1). All but three abrupt cooling events occur for global mean temperature increases below the often invoked 2 °C limit, in line with a recent study showing the high occurrence of oceanic transitions for moderate levels of global warming^45^.

**Figure 3:**
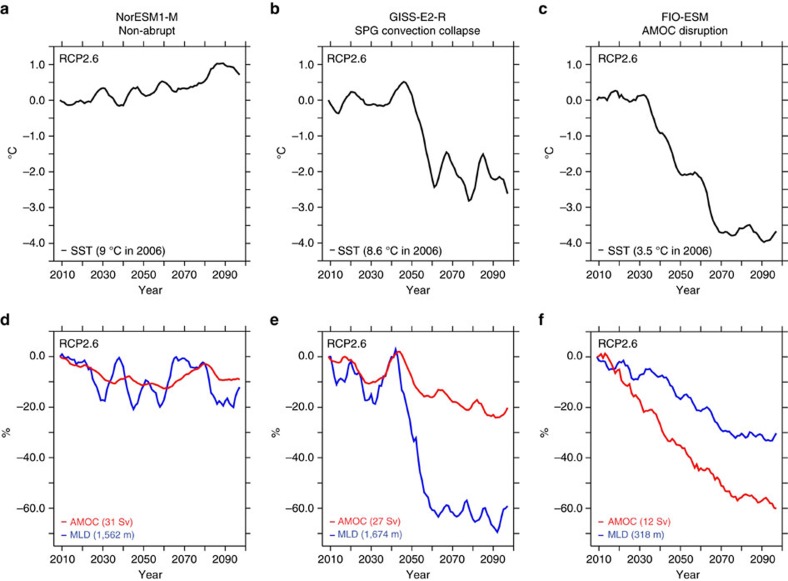
Characterization of the different SST responses in the SPG. Examples of the SST, MLD and AMOC evolutions over the SPG in the three model subsets (non-abrupt, SPG convection collapse and AMOC disruption) for the RCP2.6 scenario. Only one example for each sub-ensemble is shown while the Supplementary Figs 2–6 provides a more comprehensive illustration. All time series were smoothed using a 10-year running mean to remove the high-frequency variability. (**a**–**c**) SST anomaly (^o^C) with respect to its initial magnitude, that is, the mean over the decade 2006–2015, in (**a**) NorESM1-M, that is, non-abrupt model, (**b**) GISS-E2-R, that is, SPG convection collapse model, (**c**) FIO-ESM, that is, AMOC disruption model. Values in brackets indicate SST magnitudes at the beginning of the RCP2.6 experiments (2006–2015). (**d**–**f**) Relative changes (%) of AMOC (red lines) and MLD (blue lines) in **d** NorESM1-M, (**e**) GISS-E2-R, (**f**) FIO-ESM with respect to their initial values (2006–2015). Absolute magnitudes of AMOC (Sv) and MLD (m) averaged over the period 2006–2015 are, respectively, displayed in red and blue brackets. It is worth noticing that the strong AMOC reduction in the FIO-ESM model already takes place during the historical period (Supplementary Fig. 6), yielding a low absolute value over the 2006–2015 period.

In Fig. 3, an example of the different features characterizing each subset of models is shown for the RCP2.6 scenario, while a more comprehensive illustration and discussion is provided in Supplementary Figs 2–6 and Supplementary Notes 1–2. In order to clearly identify the abrupt signal, we smoothed all time series by applying a 10-year running mean, thus removing the higher-frequency internal variability. In the non-abrupt model, the SPG is characterized by a warming trend (Fig. 3a), and the AMOC and the MLD in the last decade (2091-2100) appear reduced by about 10% compared with their value over the decade 2006-2015 (Fig. 3d).

A sudden SST decrease of around 3 °C in 10 years typifies the SST response in the SPG convection collapse model (Fig. 3b). It occurs in combination with a sudden contraction of the MLD, which appears more than halved in 2091–2100 (Fig. 3e). The SST drop is also preceded by a rapid SPG freshening, which leads to an abrupt sea surface density decline responsible for the local MLD reduction and the associated decrease in vertical heat fluxes (Supplementary Figs 2 and 3). Although an SPG convection collapse weakens the AMOC, the latter does not collapse but experience a relatively limited and linear reduction, contrary to the non-linear response of both the MLD and the SST (Fig. 3e and Supplementary Fig. 4). The AMOC strength (maximum index) remains always higher than 13 Sv for all the experiments performed with SPG convection collapse models, consistent with an active deep convection in the Nordic Seas (Supplementary Fig. 5), which still sustains the overturning circulation^46^. Moreover, the AMOC change at the time of the abrupt cooling event is comparable to former AMOC variations that do not coincide with any rapid cooling (Fig. 3e and Supplementary Fig. 4), thus suggesting that the associated decrease in northward heat transport^24^,^25^ is not decisive in driving the temperature drop. Rather, the rapid cooling is mainly caused by a suddenly reduced vertical heat transfer from the deep to the upper ocean due to a collapsed convective mixing^36^.

The strong cooling observed in the AMOC disruption model exceeds 4 °C at the end of the 21st century (Fig. 3c). This subset of models exhibits a massive AMOC decline of 60% (80% if compared with its pre-industrial strength), which strongly differs from the characteristic AMOC reduction in both SPG convection collapse and non-abrupt models (Fig. 3 and Supplementary Fig. 6). The resulting negative SST anomaly involves both the SPG and Nordic Seas, spanning the entire region of deep-water formation, which is, however, unrealistically reproduced in AMOC disruption models (Supplementary Fig. 5).

**Figure 4:**
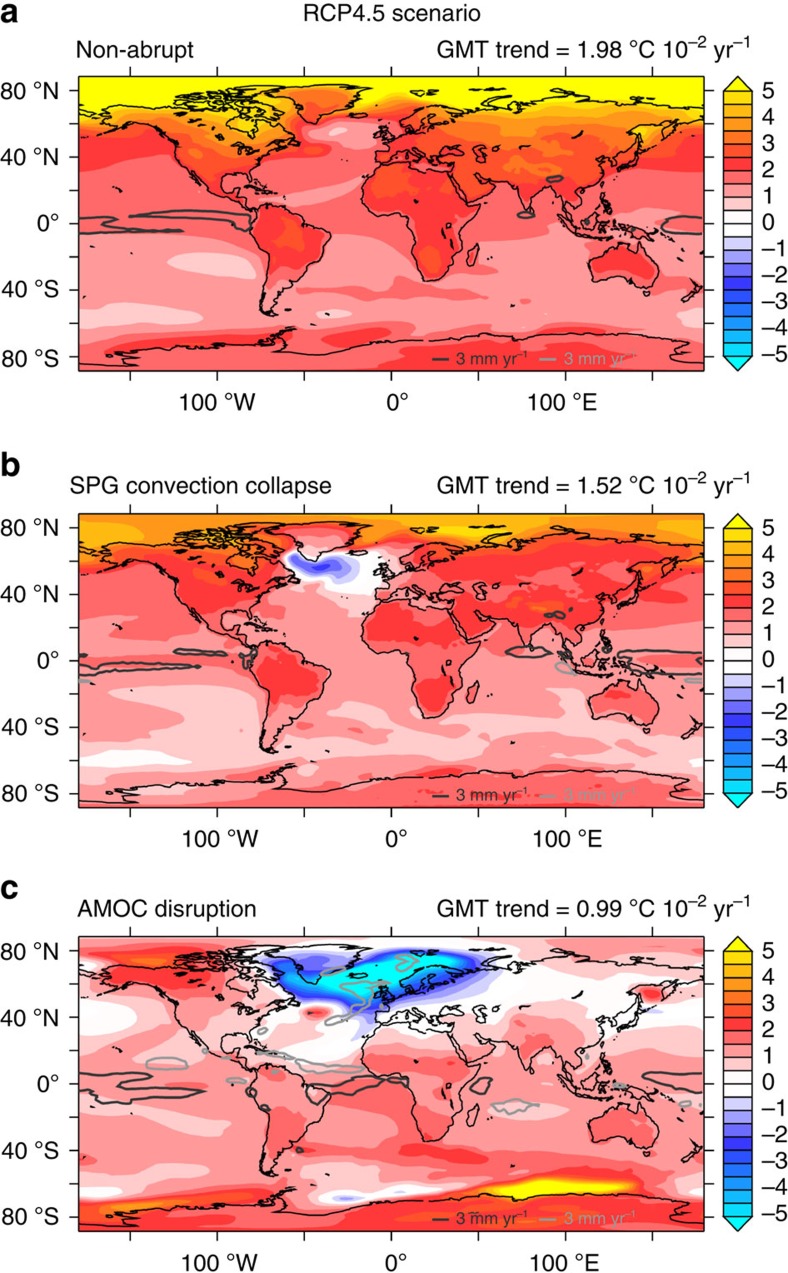
Different climate impacts. Patterns of the 21st century SAT trend (^o^C 10^−2^ year^−1^) under the RCP4.5 scenario for: (**a**) non-abrupt ensemble (27 members), (**b**) SPG convection collapse ensemble (7 members) and (**c**) AMOC disruption ensemble (2 members). The GMT trend is also displayed for each subset. The light grey and dark grey contours define regions where the ensemble mean precipitation trend, respectively, exceeds 300 mm per century and is lower than −300 mm per century. Results for the RCP2.6 and RCP8.5 scenarios can be found in Supplementary Fig. 7 and Supplementary Fig. 8.

The three different SST responses over the SPG strongly characterize three different climatic impacts. Figure 4 shows the ensemble mean surface air temperature (SAT) trend for the RCP4.5 scenario in the different subsets of models. For the non-abrupt sub-ensemble, the increase in SAT covers the whole globe (Fig. 4a), causing a global mean air temperature (GMT) trend of about 2 °C per century. The SPG convection collapse sub-ensemble shows an atmospheric ‘warming hole’ over the NA, which strongly influences the temperature response over highly populated areas such as the eastern North American coast and Western Europe (Fig. 4b), where the global warming trend is suddenly halted. The resulting GMT trend is about 1.5 °C per century. For the two models projecting a massive AMOC reduction (Fig. 4c), the northern hemisphere cools while the southern hemisphere strongly warms, consistent with the so-called bipolar seesaw^47^,^48^. The altered hemispheric temperature gradient also affects the precipitation patterns by shifting the position of the intertropical convergence zone, in line with previous findings^49^,^50^. The GMT rise in the AMOC disruption sub-ensemble is about 1 °C. However, the different levels of global warming among the models might also depend on the climate sensitivity of the CMIP5 models, for example, cloud parameterizations^51^. Qualitatively similar results were found for RCP2.6 and RCP8.5 scenarios (Supplementary Figs 7 and 8).

### The SPG stratification as a constraint for SST projections

**Figure 5:**
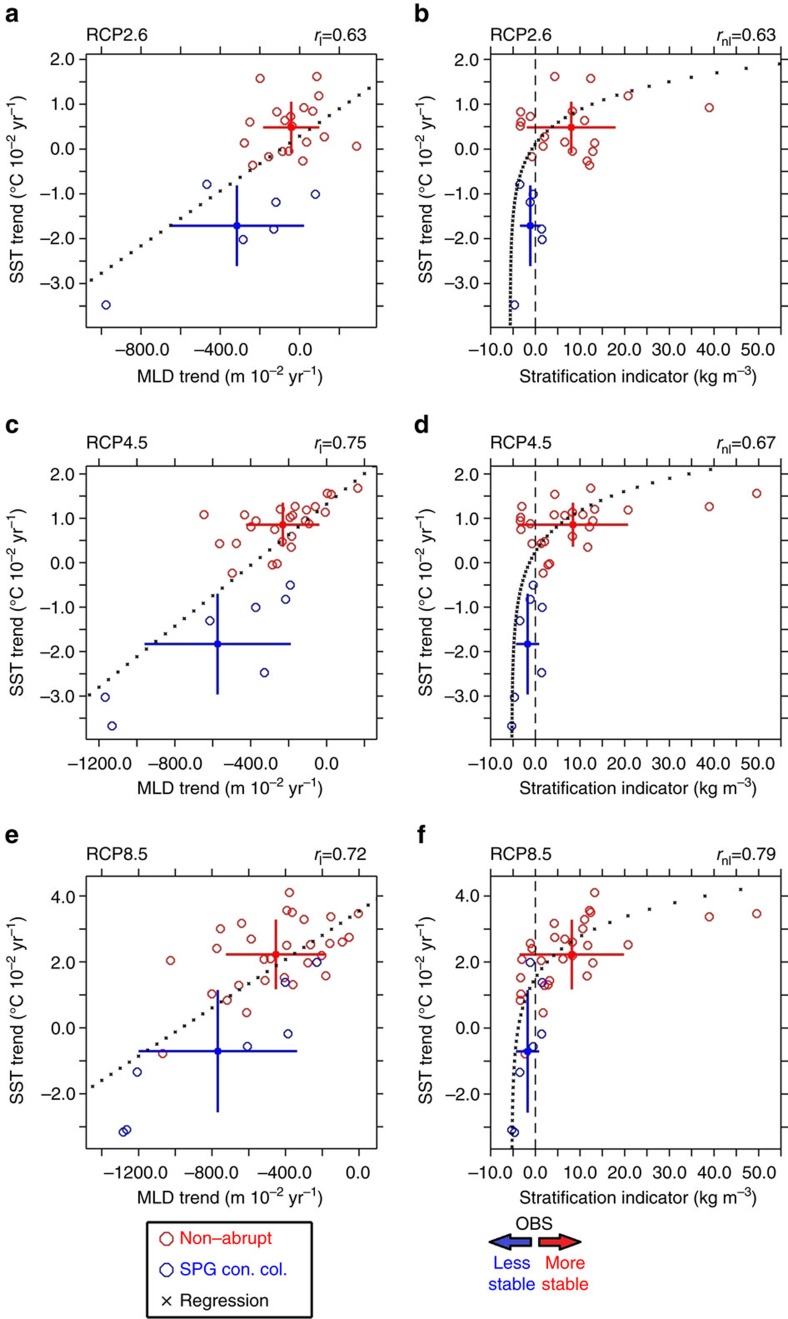
The role of stratification in SPG projections. Scatterplot of simulated SST trends (^o^C 10^−2^ year^−1^) over the SPG versus (**a**,**c**,**e**) the relevant MLD-trend (m 10^−2^ year^−1^) and (**b**,**d**,**f**) the present-day stratification indicator (Kg m^−3^). Non-abrupt models are indicated with red circles and SPG convection collapse models with blue circles, for (**a**,**b**) the RCP2.6, (**c**,**d**) the RCP4.5, (**e**,**f**) the RCP8.5 scenario. In **a**,**c**,**e** the value *r*_l_ indicates the linear correlation between the SST and MLD trends, whose significance above the 95% confidence level was evaluated with a two-tailed Student’s *t*-test. The crosses indicate the linear best-fit of the SST trends against the MLD trend, that is,. the linear regression using the least squares method. In **b**,**d**,**f** the value *r*_nl_ indicates the non-linear correlation between SST-trend and the stratification indicator, statistically significant at the 95% confidence level (see Methods). The crosses indicate the logarithmic best-fit of the SST trends against the stratification index, that is, the logarithmic regression using the least squares method. The dashed vertical black line centred on 0 indicates the observationally based stratification index, calculated as the average of GLORYS Reanalysis (1993–2012) data and EN3 analysis data (1950–2012). The arrows at the bottom indicate the areas in the panels for which the simulated SPG stratification is either more, or less stable than in the observational data.

The model spread in SST projections over the SPG stems from different dynamical responses in the convective regions. Excluding the two AMOC disruption models (FGOALS-s2 and FIO-ESM), for which an extended analysis including MLD changes over Nordic Seas would be required, the MLD response in the SPG is crucial in determining the SST evolution in the subpolar NA. The panels on the left in Fig. 5 show the linear correlation between the simulated SST trend and winter MLD trend, which ranges from 0.63 to 0.75 for the different scenarios. A shallower MLD causes the surface heat loss to the atmosphere to be less well counterbalanced by upward mixing of heat from deeper layers^37^. This implies that changes in the vertical density profile have a key role. In particular, the importance of the modelled present-day SPG winter density stratification (hereafter named background stratification, see Methods) in constraining the future SST evolution in the SPG is evidenced in the panels on the right in Fig. 5. The relation between background stratification and SST projection is non-linear and becomes more robust for more severe warming scenarios. The non-linear correlation (Methods), significant at the 95% confidence level, ranges from 0.63 for RCP2.6 simulations to 0.79 for RCP8.5 simulations. Models projecting an abrupt SPG cooling are characterized by weaker background stratification, while models featuring a more stratified SPG are more prone to project a continuous warming trend. This is physically robust since stronger (weaker) stratification is symptomatic of weaker (stronger) convective activity, and, therefore, the potential for cooling effects due to a MLD reduction is lower (higher). The model uncertainty in projecting SST over the SPG is, therefore, significantly related to the spread in simulating the winter vertical density profile for present-day conditions. This makes the background stratification a promising ‘emerging constraint’^52^ for the future SST evolution in the SPG. These conclusions do not change if a more restricted area for the calculation of the MLD and the stratification is used (Supplementary Fig. 9).

**Figure 6:**
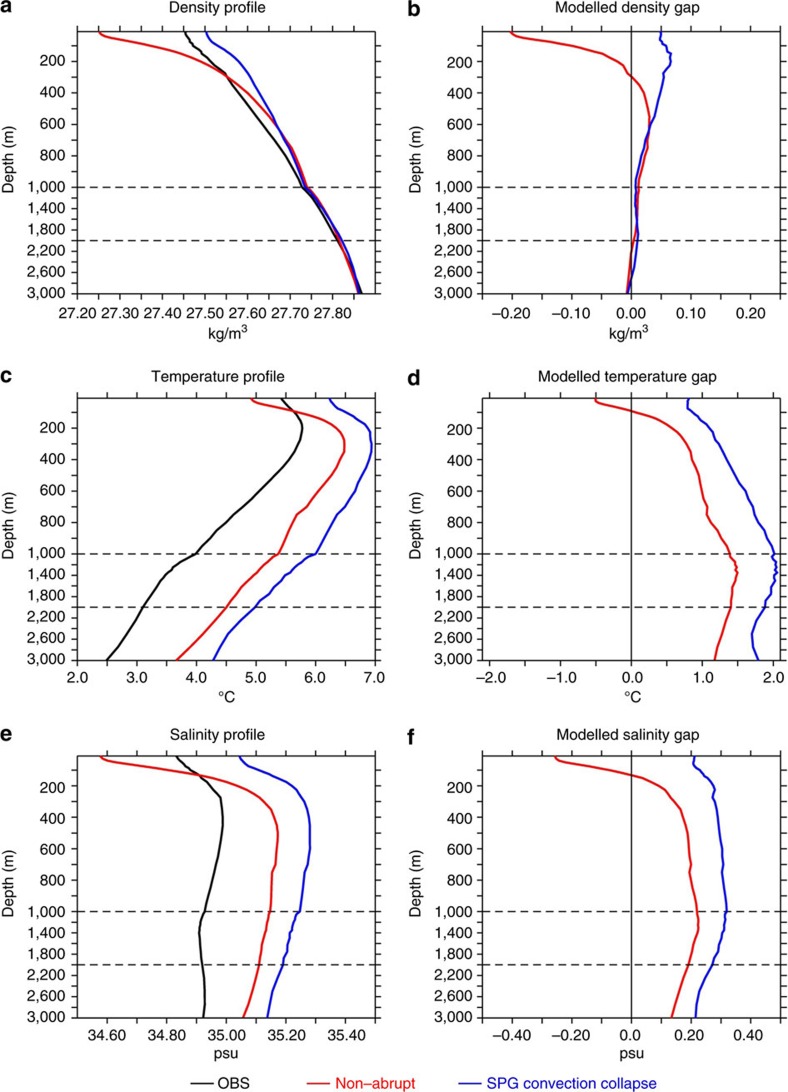
Different SPG stratifications in the model sub-ensembles and their comparison with the observational data. Present-day vertical profiles of (**a**) winter density (kg m^−3^), (**c**) temperature (^o^C) and (**e**) salinity (psu) in the SPG region for observational data (black lines), for ensemble-mean of non-abrupt models (red lines) and for ensemble-mean of the SPG convection collapse models (blue lines). Right panels show the difference between the modelled present-day winter conditions in the SPG and the observation-based data (**b**) for density, (**d**) for temperature and (**f**) salinity. Horizontal dashed lines are drawn at every 1,000 metre. A zoom has been made for the first 1,000 metres.

The right panels of Fig. 5 also show that the CMIP5 ensemble is biased towards a too stratified SPG for present-day conditions, and that, on average, the background stratification in SPG convection collapse models compares better with the observations than that in non-abrupt models. The difference in mean background stratification between the non-abrupt sub-ensemble and the SPG convection collapse sub-ensemble is significant at the 95% according to a Monte Carlo test (Supplementary Fig. 10). This difference is further detailed in Fig. 6 where the vertical profiles of winter density, temperature and salinity for present-day conditions in non-abrupt and SPG convection collapse sub-ensembles are compared with observational data. The non-abrupt ensemble is on average excessively stratified in the SPG. This is a direct consequence of an excessively low density in the upper ocean, which is mainly due to a negative salinity bias, only partly compensated by a cold SST bias. The too strong stratification limits the MLD and deep-water formation. By contrast, the SPG convection collapse ensemble is, on average, much less stratified in the SPG, rendering the region more suitable for a deep winter mixed layer. This makes the cooling effect due to a MLD reduction potentially more effective than in the non-abrupt ensemble. The fact that a large number of non-abrupt models significantly overestimate the present-day SPG stratification (Fig. 5) suggests that a tipping point for the local convection collapse cannot exist for them, as an already too weak present-day convective activity prevents any future abrupt shift to a collapsed state. This implies that the chance of future abrupt cooling events in the NA may be underestimated when considering the whole CMIP5 model ensemble.

### The reliability of the different SST projections over the SPG

Since the spread in SST responses over the SPG can be linked to different model biases, it follows that not all future climate projections are equally plausible, bringing model reliability into question. Our approach to evaluate this reliability consists of assessing the model’s capability to reproduce a relevant observable metric^52^. Here we use the background stratification as a ‘performance metric’, given its relevance in constraining SST projections over the SPG, proven in Fig. 5. We also tested whether the present-day AMOC may act as an emerging constraint. However, it turns out that there is no robust statistical relation between the simulated present-day AMOC and future SST trends over the SPG across the CMIP5 ensemble (Supplementary Fig. 11).

**Figure 7:**
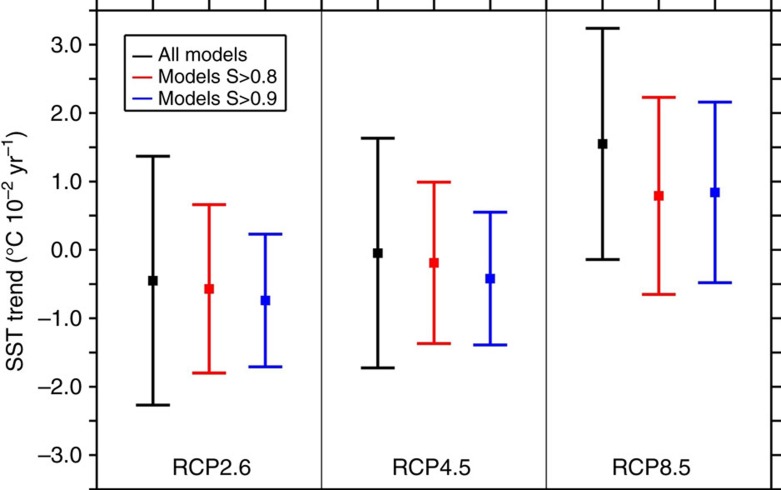
Reduction of model uncertainty over the SPG. Model ensemble mean and spread of the 21st century SST trend (^o^C 10^−2^ year^−1^) over the SPG in the RCP scenarios for different subsets of models: (black) all the 40 CMIP5 models; (*red*) CMIP5 models possessing a skill score *S*>0.8; (blue) CMIP5 models possessing a skill score *S*>0.9. Error bars indicate the standard deviation of the SST trend ensemble mean for the different subsets of models.

For each model, we computed a skill score *S* that measures the model’s accuracy in reproducing the observed present-day winter density profile over the SPG (Methods). The values of *S* across the CMIP5 models (Supplementary Table 1) range between 0, which corresponds to an extremely unrealistic reproduction of the background stratification, and 1, which corresponds to a simulated background stratification perfectly matching the observations. The skill score allows a selection of models for a more reliable analysis. By setting *S*=0.8 (0.9) as an acceptable limit for model credibility in its representation of the SPG stratification, the ensemble of models surpassing this limit consists of 18 (11) members. Such a model selection reduces the spread in SST projections over the SPG originally exhibited by the 40 CMIP5 models (Fig. 7). Moreover, the most skilled models clearly produce a more moderate SPG warming trend for the RCP8.5 scenario and a cooling trend for the RCP2.6 scenario and the RCP4.5 scenario (Fig. 7). This is linked to the strong MLD reduction under the RCP scenarios evidenced by all the most skilled models, independent of the occurrence of an SPG convection collapse. The MLD reduction induces a local cooling opposing to the global warming, which may regionally result in a subdued warming or even cooling.

The analysis of the most reliable models also highlights that the likelihood of an SPG convection collapse increases for models featuring a better background stratification. The probability of occurrence of a SPG convection collapse is 17.5% when all models are considered, that is, seven models over 40. However, the probability becomes 33.3% (45.5%) if only the 18 (11) models possessing a skill score *S*>0.8 (0.9) are considered (Table 2). Similarly, by weighting the CMIP5 models by their skill scores (Method), the likelihood of a future SPG convection collapse becomes 26.6%. This is a direct consequence of the fact that the SPG convection collapse ensemble features the best skill score among the three subsets of models.

These results highlight that the potential occurrence of an SPG convection collapse in CMIP5 models is conditional on a realistic representation of the local background stratification. However, this does not imply that there exists a deterministic relation between background stratification and convection collapse, since 12 (6) of the 18 (11) most skilled models do not project any abrupt event in the SPG. Convection generally depends on the stratification, but details in the particular configuration of temperature and salinity are also important, notably for SST and Sea Surface Salinity (SSS)^53^. A common feature of the 12 (6) non-abrupt models possessing a skill score *S*>0.8 (0.9) is that they simulate, on average, too warm and salty SPG surface water masses for present-day conditions, that is, SST=6.9±1.2 °C (6.4±1.2 °C) and SSS=35.1±0.4 psu (35.1±0.3 psu), as compared to observations, that is, SST=5.4±0.3 °C and SSS=34.8±0.0 psu. This configuration differs strongly from that in the remaining non-abrupt models, which are, on the contrary, too cold and fresh, that is, SST=3.4±1.9 °C (4.4±2.4 °C) and SSS=34.1±0.6 psu (34.4±0.7 psu). The SPG convection collapse ensemble features the smallest bias in SST and SSS, that is, SST=5.9±0.9 °C (5.8±0.9 °C) and SSS=35.0±0.1 psu (35.0±0.2 psu), and their models would have been estimated the most reliable also by using a multi-parameter skill score based on SST and SSS. These different configurations, consistent with the density compensating SST and SSS biases already evidenced across the CMIP5 models^54^, further explain the different responses among the models. Indeed, the deep-water formation is sensible to SST and SSS, with a warmer/saltier configuration being more favourable for deep convection than a colder/fresher configuration^53^. Hence, in models reproducing too cold and fresh SPG surface water masses for present-day conditions (that is, non-abrupt models with *S*<0.8), the convective activity may already be unrealistically inhibited before global warming, suggesting the non-existence of a climatic threshold for an abrupt convection collapse. On the contrary, in models that are biased towards too warm and salty SPG surface water masses for present-day conditions (that is, non-abrupt models with *S*>0.8), the climatic threshold for a transition to a collapsed convection potentially exists, but its achievement might be unrealistically long postponed.

## Discussion

The paradigm that the potential for NA abrupt changes mainly depends on the fate of the AMOC is clearly incomplete. In addition to the potential existence of a tipping point for an AMOC shutdown, we argue that a separate one involving a collapse of SPG convection^46^ also exists. Both AMOC disruption and SPG convection collapse are possible responses to the ongoing global warming trend^43^,^44^ and changes in the hydrological cycle that are freshening the northern NA^31^,^55^. However, while the risk of an AMOC shutdown has been largely debated^24^,^30^, an assessment of the possibility of a local SPG convection collapse and its potential impacts was missing so far. Our results highlight that in CMIP5 models the occurrence of a NA abrupt cooling due to an SPG convection collapse is almost four times more likely than the occurrence of a NA abrupt cooling due to an AMOC disruption. Furthermore, when considering only the most realistic models in simulating the present-day SPG stratification, the chance of an NA abrupt cooling in the coming century is close to 50%, while the chance of a complete AMOC collapse is negligible.

The separation between SPG convection collapse and AMOC disruption abrupt events does not mean that SPG deep-water formation and the AMOC are independent. Since the local SPG convection is part of the large-scale overturning circulation system, an interruption of SPG deep-water formation does weaken the AMOC. However, for an AMOC disruption to occur, deep-water formation has to collapse at all sites where it occurs. The abrupt events detected in SPG convection collapse models are associated with a suspension of local deep-water formation, but do not coincide with a dramatic AMOC decline because convection is sustained (or even reinforced) at other locations. Rather, the separation between SPG convection collapse and AMOC disruption abrupt events stems from their different dominant drivers, which differentiate the characteristic timing and spatial extent of these events. On the one hand, the SPG temperature drop of around 2 °C in SPG convection collapse models takes place in less than a decade, and is mainly due to a local convective feedback causing an interruption of the vertical heat transfer from the deep ocean to the surface. The occurrence of such an abrupt shift in seven models suggests the potential bistability of the SPG^31^, in agreement with a recent study based on an evaluation of CMIP3 models^32^. On the other hand, the abrupt cooling in AMOC disruption models is more linear and occurs over a longer timescale. It is mainly due to a large-scale advective feedback causing a long-lasting reduction of the ocean’s northward heat transport, which drives a more regular but persistent temperature decrease over the entire northern NA, that is, up to 4 °C in 50 years. This confirms the large inertia of the AMOC in CMIP5 models^56^, for which a shutdown has been previously shown to occur through a gradual decline^57^ rather than in an abrupt fashion as suggested in conceptual models^22^,^24^ and models of intermediate complexity^58^.

A central point of our analysis is that a realistic present-day stratification over the SPG is a necessary requirement for an abrupt SPG convection collapse to occur in a model. Our argument is based on the finding that the present-day stratification in the SPG acts as an emerging constraint for the local temperature response. The large uncertainty in SST projections shown by CMIP5 experiments indeed reflects the wide inter-model spread in representing the SPG background stratification. While all the SPG convection collapse models feature a relatively realistic SPG stratification for present-day conditions, the ensemble of CMIP5 models, as a whole, is biased towards a too stratified SPG. We, therefore, assess that the ensemble of CMIP5 models underrepresents the possibility of an SPG convection collapse, thus corroborating the view that current generation climate models may be too stable^59^. Further support for our assessment is provided by the fact that melt-water and iceberg discharges from the Greenland ice sheet were not accounted for in CMIP5 projections. These processes may further inhibit the deep-water formation in the SPG through freshening of the surface waters^60^, thus potentially increasing the chance of a local convection collapse in the future. To obtain a more reliable assessment of this risk, the next generation of climate models should incorporate freshwater forcing related to ice-sheet melting and calving. Simultaneously, the model ensemble spread should be narrowed down over the NA, notably by reducing the model biases in reproducing a realistic SPG stratification.

It is worth stressing that although a necessary condition, a reliable simulation of the SPG present-day stratification is not a sufficient condition for the onset of an SPG convection collapse in model projections, since a few models featuring a realistic background stratification do not project such an event. Climate transitions strongly depend on the stochastic combination of internal dynamics and external forcing^61^. Thus, the relatively short RCP simulations might have been insufficient for the onset of a rapid transition in certain models. Also, details in local SST and SSS, climate sensitivity, and the amplitude of the atmospheric noise can all be decisive in creating the abrupt event, making the latter essentially a chance process to be assessed through a likelihood of occurrence across the CMIP5 models. A main conclusion of this study is that such a chance notably increases when considering only the most skilled models in reproducing a realistic SPG background stratification. Furthermore, regardless of the occurrence of a convection collapse, the most skilled models project a much more moderate warming or even cooling trend over the NA than the CMIP5 ensemble-mean. The occurrence of this NA warming hole in models is always associated with a large decrease in MLD over the SPG, which is decisive in producing such a response. This opens up a new interpretation of the cause of the observed 20th century cooling over the NA^10^,^11^,^38^, which up to now has been mostly related to an AMOC weakening^20^. The fact that the observed cooling is mainly centred over the Labrador and Irminger Seas reinforces our view of an underlying local process involving SPG convection rather than large-scale AMOC changes.

A final issue concerns the climate impact that a convection collapse in the SPG has on the surrounding regions. The repercussions of such an event on temperature and precipitation represent an important hazard for many economic sectors, notably for the agriculture industry as well as for water resources and energy management. Also, the associated modifications in ocean circulation alter the distribution of the main faunistic zones over the northern NA^62^, with strong implications for the fishery sector. Our analysis suggests that these potential risks are erroneously underrated. For instance, over the UK, the temperature evolution in SPG convection collapse models largely deviates from the continuous warming trend characterizing the CMIP5 ensemble-mean, even exceeding the CMIP5 ensemble-mean standard deviation. When only looking to the CMIP5 ensemble mean, such a discordant temperature projection over the UK would appear as an extreme case and very unlikely to occur. However, our assessment, discussed in this paper, is that the chance for such a discordant response over the UK is actually almost as large as the chance of a continuous warming trend. Given the recent evidence of reversed climatic trends over the NA^63^ and the impact that the current Greenland meltwater accumulation may have on Labrador Sea convection^64^, we ultimately stress the need to consider the potential risks associated with an SPG convection collapse when developing future strategies of adaptation to climate change as well as when searching for possible early warning signals^65^ of abrupt climate change in the Atlantic. Since the AMOC may not be primarily responsible for abrupt cooling events in the NA, observing the long-time evolution of the SPG stratification appears very relevant in light of the present results. The ARGOS and OSNAP programs associated with various decadal prediction systems will provide key information in the coming years to better estimate the possibility of NA rapid cooling.

## Methods

### Determination of the reference region

The definition of the region selected for our analysis is based on the SST response in the model ensemble under different forcing scenarios. For each simulation we calculated the SST trend by means of a least squared regression. Depending on the scenario, we determined maps of the ensemble mean SST-trend, that is, averaged over all members, as well as maps of ensemble spread in SST-trend, that is, the standard deviation among the members. Hence, we systematically isolated those regions where, for all scenarios, a subdued SST warming trend is observed, and the model uncertainty clearly exceeds the global mean uncertainty, using the following criteria:


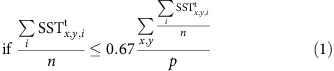



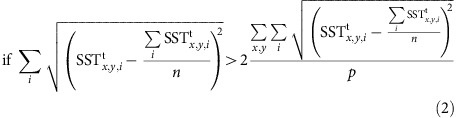


where SST^t^ is the surface temperature trend, the subscript *i* indicates the member of the model ensemble, *n* is the number of models, and the subscripts *x,y* are the longitude, latitude coordinates of the *p* points on the model grid. The constant *c*_1_=0.67 and *c*_2_=2 have been arbitrarily chosen, according to the restrictions *c*_1_<1 and *c*_2_>1.

We also defined an alternative reference region as that region for which the MLD>1,000 m according with GLORYS^13^ reanalysis data. To test the sensitivity of our conclusions upon the particular choice of the reference region we replicated key analyses of this study by calculating modelled MLD trends, stratification indicators and model skill scores over this alternative reference region without finding significant changes (Supplementary Fig. 9).

### Identification and classification of abrupt cooling events

The SST evolution in the SPG was calculated for 106 time series, in which historical simulations were merged with each RCP scenario run. Moreover, for each model we calculated the SST standard deviation over the same region from the last 300 years of the pre-industrial control simulation. This value, which ideally represents the unforced (or internal) SST variability for each model, is then used to scale the corresponding historical+RCP time series. The resulting non-dimensional signal indicates the extent to which an SST anomaly is determined by external forcing. An abrupt cooling in the SPG region was defined as an SST drop for which the 10-year normalized anomaly is larger than three. Under a Gaussian assumption, this means that there is a probability of <0.3% that an SST anomaly of the same magnitude already occurred in the pre-industrial control simulation.

### The AMOC index and the winter MLD

The AMOC index was defined as the maximum of the zonally integrated Atlantic overturning streamfunction (on y-z plane) between 30°S and 60°N and between 500 m and the bottom. A different AMOC index calculated at 26°N has also been used without making a significant impact on the results (Supplementary Fig. 11).

The MLD indicates the thickness of the upper ocean that directly interacts with the atmosphere. It has been determined from the (diagnostic) Mass Density *σ*_*t*_ (kg m^−3^) of seawater, derived from the (prognostic) absolute salinity S (psu), *in-situ* temperature T (^o^C) and reference pressure P (dbar), using the 1980 UNESCO International Equation of State (IES80)^66^. By following the criterion of Levitus (1982) (ref. 67), the MLD is defined as the depth *z* at which the difference between *σ*_*t*_*(z)* and *σ*_*t*_ (*0*) becomes 0.125 kg m^−3^. In relation to a given year, the winter mixed layer is then defined as the maximum MLD during the 3 months of January, February and March.

### Calculation of the non-linear correlation

The Pearson correlation between two sets of data (*x* and *y)* measures the robustness of the approximation *y=ax+b*, with *a*, *b* constants. The panels on the right in Fig. 3 clearly show that the SST trend (*x* hereinafter) and stratification index (*y* hereinafter) are proportional. However, they seem to follow a non-linear relation rather than a simple linear relation *y=ax+b*. To quantify the degree of correlation between these two metrics, while allowing for non-linearities, we adopted a procedure consisting of (*i*) assuming a non-linear relation *y=f(x)* between the variables, (*ii*) transforming the data, and (*iii*) computing the linear correlation between the transformed data. After the translation y*=y−y_*min*_, imposed to make all x data positive, we assumed an exponential relation y*=ce^*ax*^, which gives ln y*=ax+ln *c*. Through the transformation *Y=*ln y* and *X=x*, we obtain *X=aY+*ln *c*. Consequently, the calculation of the non-linear correlation between x and y can be reduced to the calculation of the linear correlation between *X* and *Y*. The significance of the non-linear correlation has been consequently tested with a two-tails Student’s *t*-test applied to *X* and *Y*. Similar values for the non-linear correlation as displayed in Fig. 3 can be found by using the Spearman’s rank correlation coefficient.

### Observation-based data

For the comparison of model results with observational data, we used GLORYS2V1 (ref. 13) reanalysis data provided by Mercator Ocean and EN3_v2a (ref. 68) analysis produced by the Met Office Hadley Centre.

GLORYS produces monthly global ocean reanalyses at eddy-permitting resolution (1/4°) from 1993 to 2012. The reanalysis is based on the ocean and sea-ice general circulation model NEMO in the ORCA025 configuration forced by surface boundary conditions derived from the atmospheric ECMWF reanalyses, and on the assimilation of *in-situ* T and S profiles as well as SST from satellite measurements and sea-level anomalies obtained from satellite altimetry.

EN3 data consist of objective analyses based on the temperature and salinity profiles derived from WOD5, GTSPP, Argo and the ASBO project. The monthly data cover the period between 1950 and 2012.

### The stratification indicator

We assigned an SPG stratification indicator *P*_*i*_ for present-day conditions to each model, where present-day refers to the mean over the period 1986–2015. Its definition consists of calculating a vertical sum of the density anomaly in the first 2,000 m for each model *i* and for the observational data OBS, and subtract these. This gives


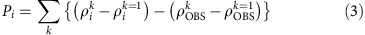


where the superscript *k* is the depth index, with *k*=1 corresponding to the surface. To enforce the increasing importance to the uppermost layers, we considered 50 different depths *z*, such that *z*^*k*^*−z*^*k−1*^ ranges from 5 m at the surface to 125 m at 2,000 m. The *P*_*i*_ index then quantifies the deviation from the observational data of the SPG stratification in each model *i*. Its sign is positive (negative) if the SPG is more (less) stratified in the model than in the observations, and its magnitude indicates how far from reality the modelled density profile is. The observational data consisted of an average between GLORYS Reanalysis data and EN3 Analysis data, but the *P*_*i*_ appeared insensitive to which observational data set was chosen. The use of *P*_*i*_ as a stratification indicator rather than others, for example, the Brunt-Väisälä frequency or MLD itself, is supported by the fact that it is not a mere measure of the stability of the water column, but it also takes into account the particular structure of the vertical density profile, which may be a further factor characterizing convective activity.

### The model’s skill score S

We exploited the stratification indicator to evaluate the model’s ability in reproducing a realistic vertical density profile over the SPG for present-day winter conditions. To estimate the reliability of the different models in projecting the SST evolution over the SPG, we defined a skill score *S*_*i*_ for each model *i*. The methodology is based on two hypotheses. We assume that (*i*) the likelihood that the model outputs are consistent with observation-based data have a Gaussian distribution^69^,^70^ identified by a probability density function PDF, with the values of the skill score *S*_*i*_ shaped by this distribution, and (*ii*) the standard deviation *σ* of the PDF is such that 67% of the stratification indicator values lie within the interval [−*σ*,*σ*]. The skill score *S*_*i*_ is, therefore, defined as


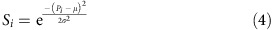


where *μ*=0, since it represents the expected value for the stratification index, which by definition coincides with the observational based data.

Moreover, the skill score *S*_*i*_ has been used for defining the SST weighted ensemble mean such that


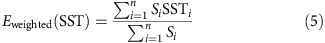


where *i* is the model index and *n* is the total number of members. Likewise, we defined a weighted likelihood of occurrence *L*_weighted_ for each type of event associated to the three subsets of models as


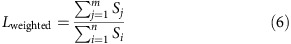


where the subscript *j* indicates the generic member of the *m* models belonging to the specific subset of models, and *i* refers to the generic member of the totality of *n* models analysed, with *m<n*.

### Code availability

All data generated during this study and the underlying Ferret codes produced for their analysis are available from the corresponding author upon request.

### Data availability

All the results supporting this study are based on CMIP5 model data, EN3 Analysis data and GLORYS Re-analysis data, which are available respectively from http://cmip-pcmdi.llnl.gov/cmip5/data_portal.html, from http://www.metoffice.gov.uk and from http://www.mercator-ocean.fr (upon request).

## Additional information

**How to cite this article:** Sgubin, G. *et al*. Abrupt cooling over the North Atlantic in modern climate models. *Nat. Commun.* **8,** 14375 doi: 10.1038/ncomms14375 (2017).

**Publisher’s note:** Springer Nature remains neutral with regard to jurisdictional claims in published maps and institutional affiliations.

## Supplementary Material

Supplementary Information Supplementary Figures, Supplementary Table, Supplementary Notes and Supplementary References

## Figures and Tables

**Table 1:** Classification of CMIP5 models in the three sub-ensembles.

Subset name	List of models			
Non-abrupt	ACCESS1-0; ACCESS1-3; bcc-csm1-1, bcc-csm1-1-m; BNU-ESM; CanESM2; CCSM4; CESM1-BGC; CESM1-CAM5-1-FV2; CMCC-CESM; CMCC-CM; CMCC-CMS; CNRM-CM; EC-EARTH; FGOLAS-g2; GFDL-CM3; GISS-E2-H; GISS-E2-H-CC; HadGEM2-AO; HadGEM2-CC; HadGEM2-ES; IPSL-CM5A-LR; IPSL-CM5A-MR; IPSL-CM5B-LR; MIROC-ESM; MIROC-ESM-CHEM; MPI-ESM-LR; MPI-ESM-MR; MRI-CGCM3; Nor-ESM1-M; NorESM1-ME			
	**Model**	**Scenario**	**Year of occurrence**	**Δ GMT (**^**o**^**C)**
SPG convection collapse				
	CESM1-CAM5	RCP8.5	∼2075	3.8
	CSIRO-Mk3-6-0	RCP2.6	∼2025	1.6
	GFDL-ESM2G	Historical	∼1920	0.2
	GFDL-ESM2M	RCP2.6RCP4.5	∼2025∼2050	1.11.9
	GISS-E2-R	RCP2.6RCP4.5RCP8.5	∼2050∼2050∼2055	1.41.61.9
	GISS-E2-R-CC	RCP4.5RCP8.5	∼2050∼2050	1.72.0
	MIROC5	RCP2.6	∼2065	1.4
				
AMOC disruption				
	FGOALS-s2	RCP2.6RCP4.5	∼2025∼2030	2.22.5
	FIO-ESM	RCP2.6RCP4.5	∼2035∼2025	1.41.6

List of models belonging to the three different sub-ensembles identified. For those models producing an SPG abrupt cooling (SPG convection collapse models and AMOC disruption models) the scenario and the year of occurrence of the event have been also displayed. In addition, the corresponding level of global warming calculated from the pre-industrial global mean temperature has been shown for all abrupt events.

**Table 2:** Implications of models’ evaluation.

Subset of models	Averaged skill score	Unweighted occurrence	Weighted occurrence	Occurrence (*S*>0.8)	Occurrence (*S*>0.9)
Non-abrupt	0.53±0.36	77.5% (31/40)	70.1%	66.7% (12/18)	54.5% (6/11)
SPG convection collapse	0.90±0.08	17.5% (7/40)	26.6%	33.3% (6/18)	45.5% (5/11)
AMOC disruption	0.39±0.30	5.0% (2/40)	3.3%	0% (0/18)	0% (0/11)

Summary of the averaged skill scores featured by the different sub-ensembles. The probability of occurrence of the event associated with each subset of models is shown, respectively, if no weighting criterion is applied to the models, if a weighting criterion is applied to the models, if only models satisfying the condition *S*>0.8 are considered, if only models satisfying the condition *S*>0.9 are considered.
