# Publisher Correction: Abrupt cooling over the North Atlantic in modern climate models

**DOI:** 10.1038/ncomms16222

**Published:** 2018-06-25

**Authors:** Giovanni Sgubin, Didier Swingedouw, Sybren Drijfhout, Yannick Mary, Amine Bennabi

Nature Communications 8: Article number: 14375; DOI: 10.1038/ncomms14375 (2017); Published online: 02 15 2017, Updated: 06 25 2018

The original HTML version of this Article omitted the article number; it should have been ‘14375’. This has now been corrected in the HTML version of the Article. The PDF version was correct from the time of publication.

