# Supplementary material for: Abrupt cooling over the North Atlantic in modern climate models
Source: Nat Commun. 2017 Feb 15;8:14375. doi: 10.1038/ncomms14375 (PMC5330854; doi:10.1038/ncomms14375)
Supplement: Supplementary Information — Supplementary Figures, Supplementary Table, Supplementary Notes and Supplementary References [file ncomms14375-s1.pdf]

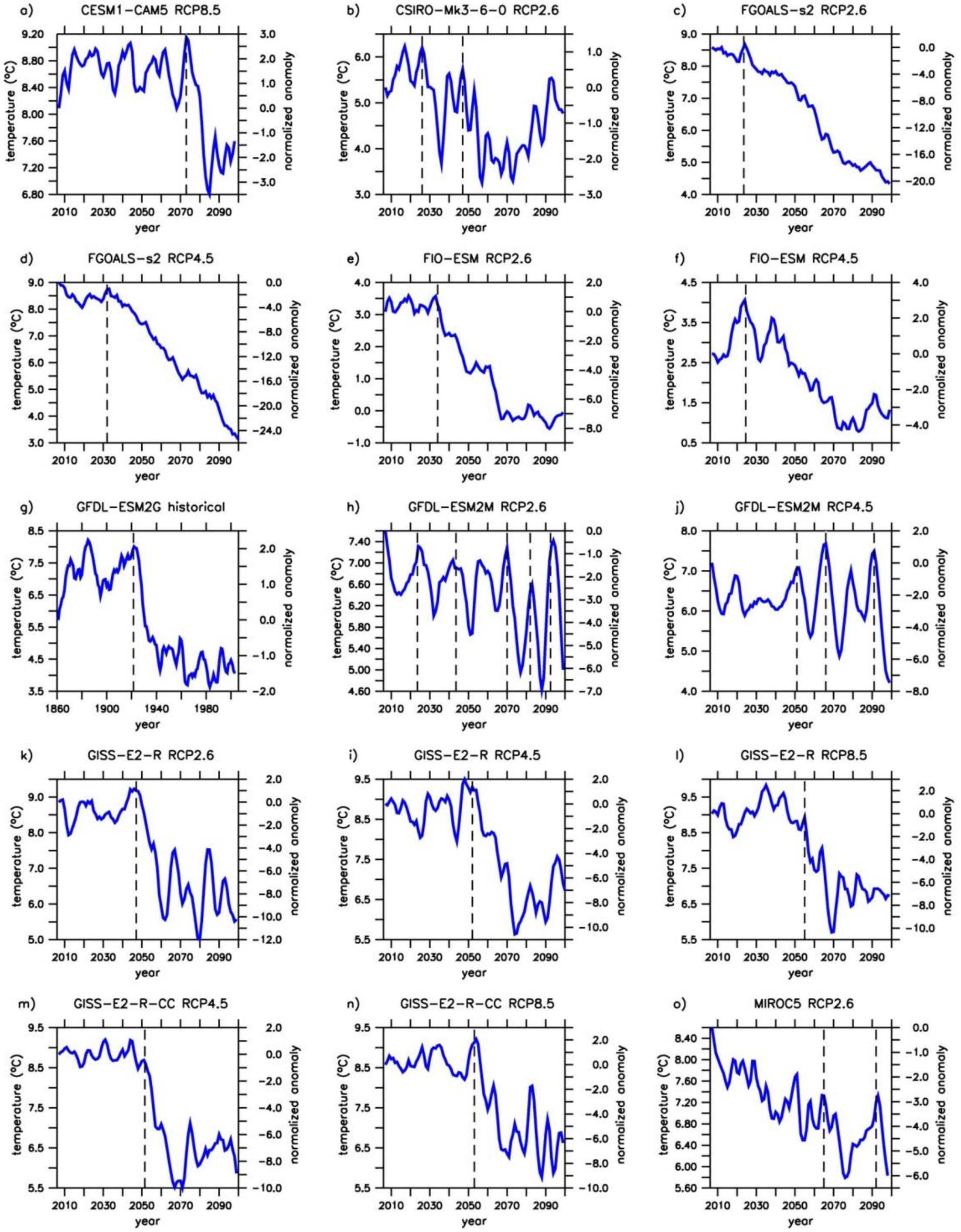


**Supplementary Figure 1. Abrupt cooling events in the SPG.** SST evolution (ºC) in the models showing an abrupt cooling. The vertical axis on the left side of the panels specifies the absolute SST, while the right-side axis specifies the SST anomaly normalized by the standard deviation of SST in piControl experiment (Methods). Vertical dashed lines indicate when an abrupt event initiates.

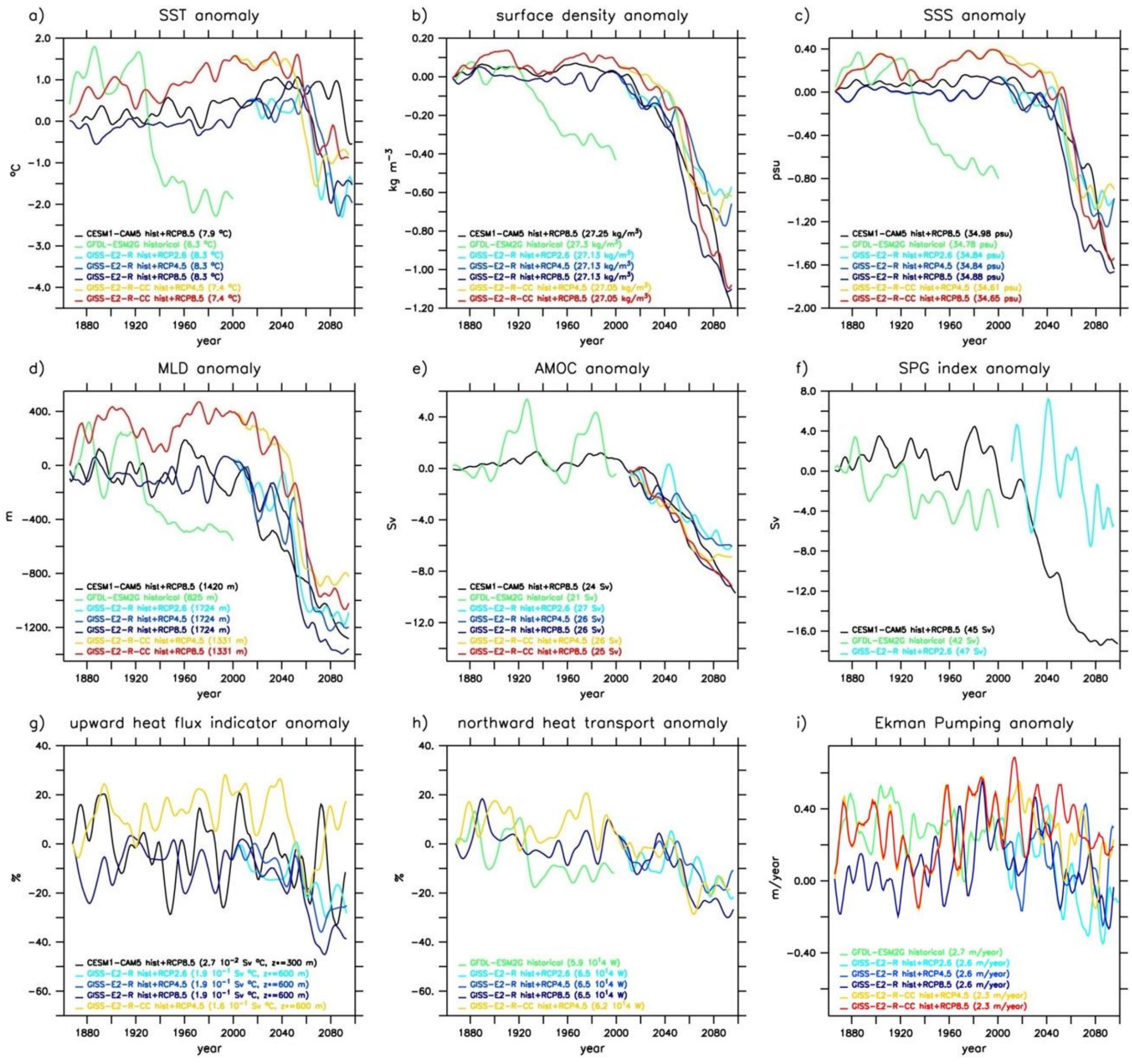


**Supplementary Figure 2. Mechanisms of abrupt cooling in the *SPG convection collapse* sub-ensemble (part I).** Evolution of the main physical parameters playing a role in the onset of a single SST drop in the SPG region. Anomalies from the main values of pi-Control simulation are shown for: (a) sea surface temperature, *i.e.* SST (°C) , (b) sea surface density (kg $m^{-3}$), (c) sea surface salinity, *i.e.* SSS (psu), (d) MLD (m), (e) AMOC (Sv), (f) subpolar gyre (SPG) index (Sv), (g) vertical heat flux indicator (Sv °C), (h) meridional northward heat transport at 48°N (W) and (i) Ekman pumping variations over the SPG (m/year). The MLD index was calculated according to the definition provided in Methods. The AMOC values refer to the maximum index defined in *Methods*. The SPG index is defined as the local minimum of the barotropic

streamfunction within the reference region. The indicator for vertical heat fluxes at depth z* is calculated as $w_{z^*}S_{z^*}T_{z^*}$ where $w_z$ is the mean vertical velocity across the SPG surface $S_z$ and $T_{z^*}$ is the mean SPG temperature at depth z*. However, such an indicator has to be considered as a mere qualitative measure of the changes in upward heat flux. Indeed it accounts for just the advective component of the heat flux without considering the diffusion, which may be also be significant. For a given metric, this analysis is limited to the available CMIP5 data.

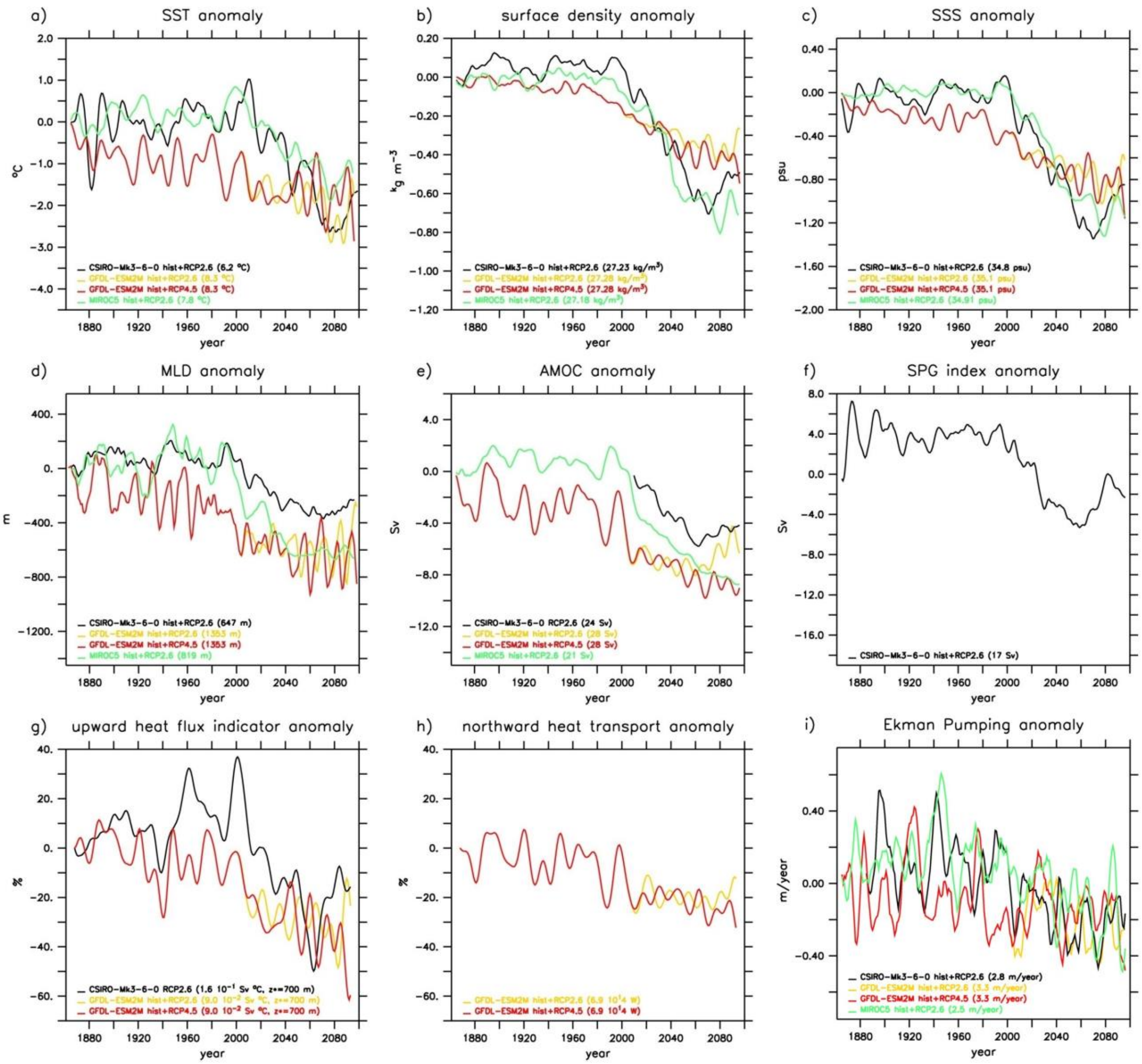


**Supplementary Figure 3. Mechanisms of abrupt cooling in the *SPG convection collapse* sub-ensemble (part II).** Same as Supplementary Fig. 2 but for SPG convection collapse models showing multiple SST drops.

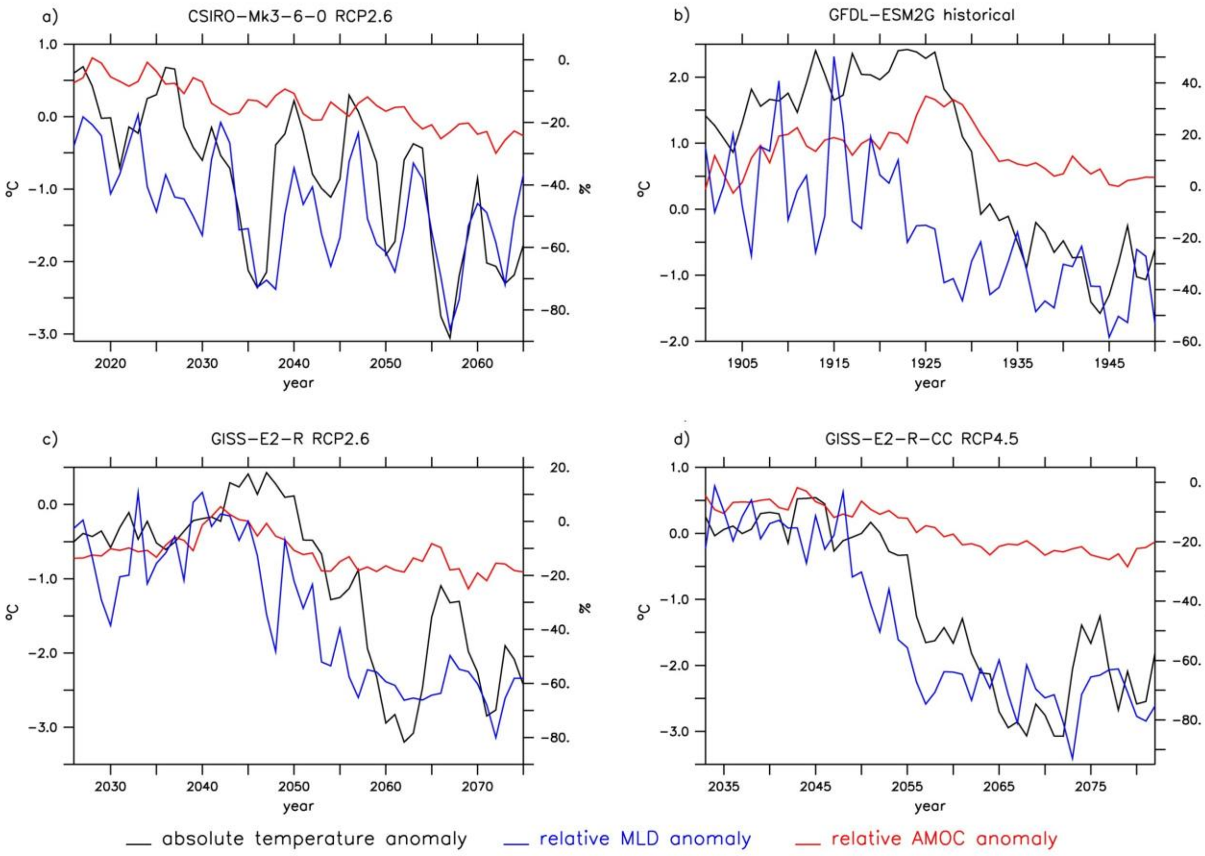


**Supplementary Figure 4. Processes at play to trigger abrupt cooling in a few models from *SPG convection collapse* ensemble.** Other examples illustrating the mechanistic link between SPG temperature drops (in °C, black line) and changes in local mixed layer depth (in % of decrease as compared to its initial value, blue line) and AMOC (in % of decrease as compared to its initial value, red line). A temporal zoom has been applied around the time of the abrupt cooling event. No smoothing has been applied and annual mean are shown. The name of the model considered is shown on top of each panel.

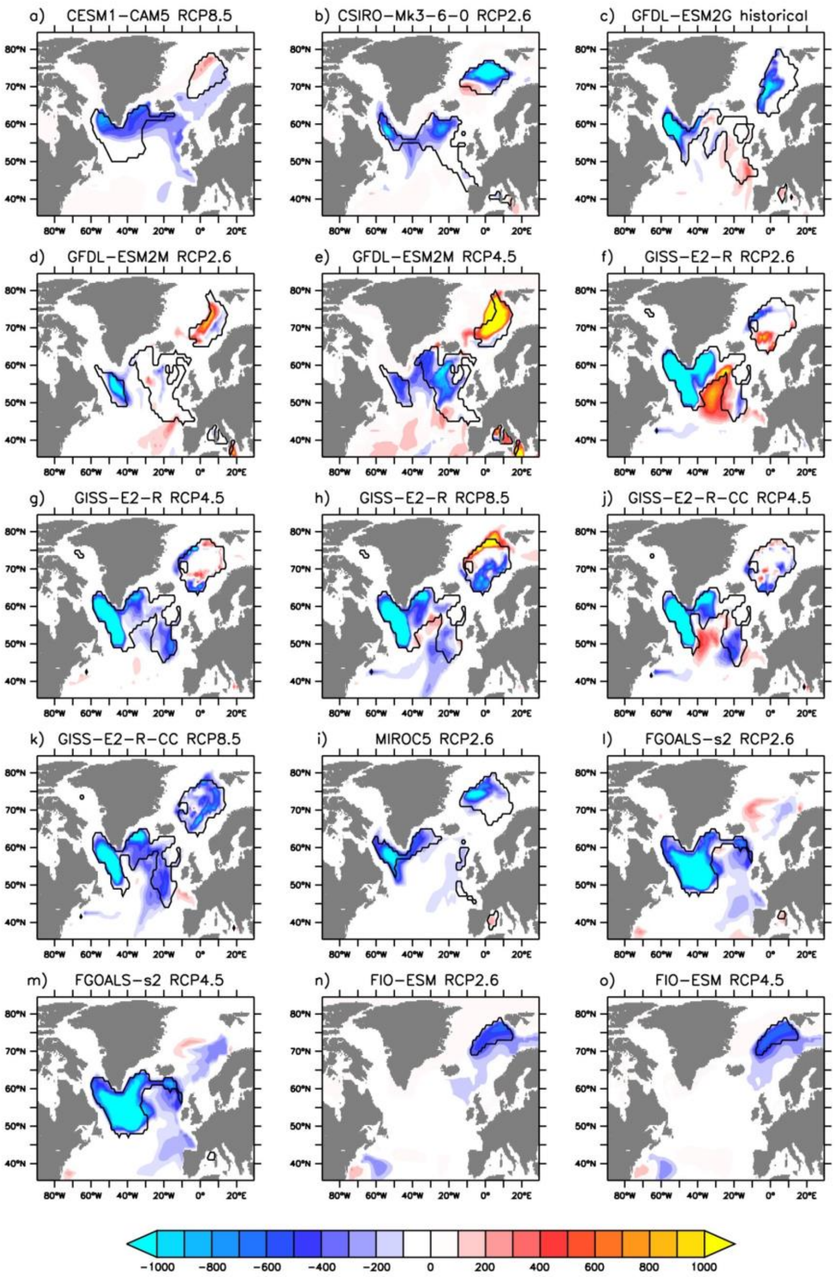


**Supplementary Figure 5. Relation between abrupt cooling events in the SPG and changes in MLD field.** Difference (m) between the mean 10-year MLD field following an abrupt cooling event and the mean 10-year MLD field preceding an abrupt cooling event. The black contour lines indicate the regions for which MLD>1000 m over the period 1986-2015, *i.e.* the sites of deep-water formation for present-day conditions in the different models. Panels from a) to i) refer to SPG convection collapse models, while panels form l) to o) refer to AMOC disruption models. Note that convective regions in AMOC disruption models are unrealistically reproduced for present-day conditions, as they consist in just one site of deep-water formation.

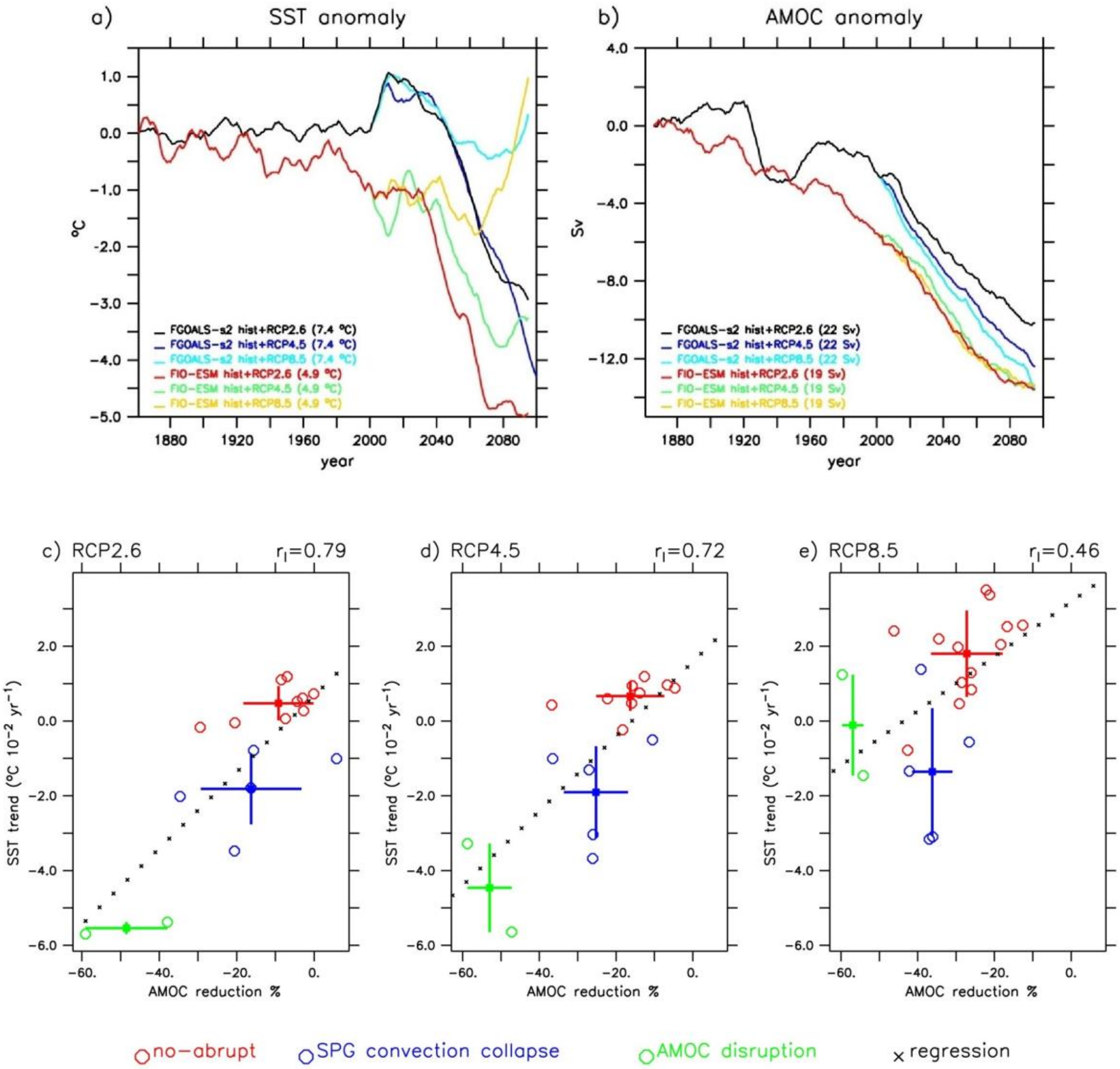


**Supplementary Figure 6. The massive AMOC reduction as a driver of the NA abrupt cooling in AMOC disruption models.** (a) Evolution of SST anomaly (°C) and b) of the AMOC anomaly (Sv) under different RCPs scenarios for the AMOC disruption models. (c-e) Scatterplot of simulated SST trends (°C $10^{-2}$ year$^{-1}$) over the SPG versus the percentage of AMOC reduction (differences between 2086-2100 values and 2006-2015 values) across the CMIP5 models for c) RCP2.6, d) RCP4.5, and e) RCP8.5 scenario. Non-abrupt models are indicated with red circles, SPG convection collapse models with blue circles, and AMOC disruption models with green circles. Note that AMOC reduction in SPG convection collapse models and non-abrupt models is much lower than in AMOC disruption models.

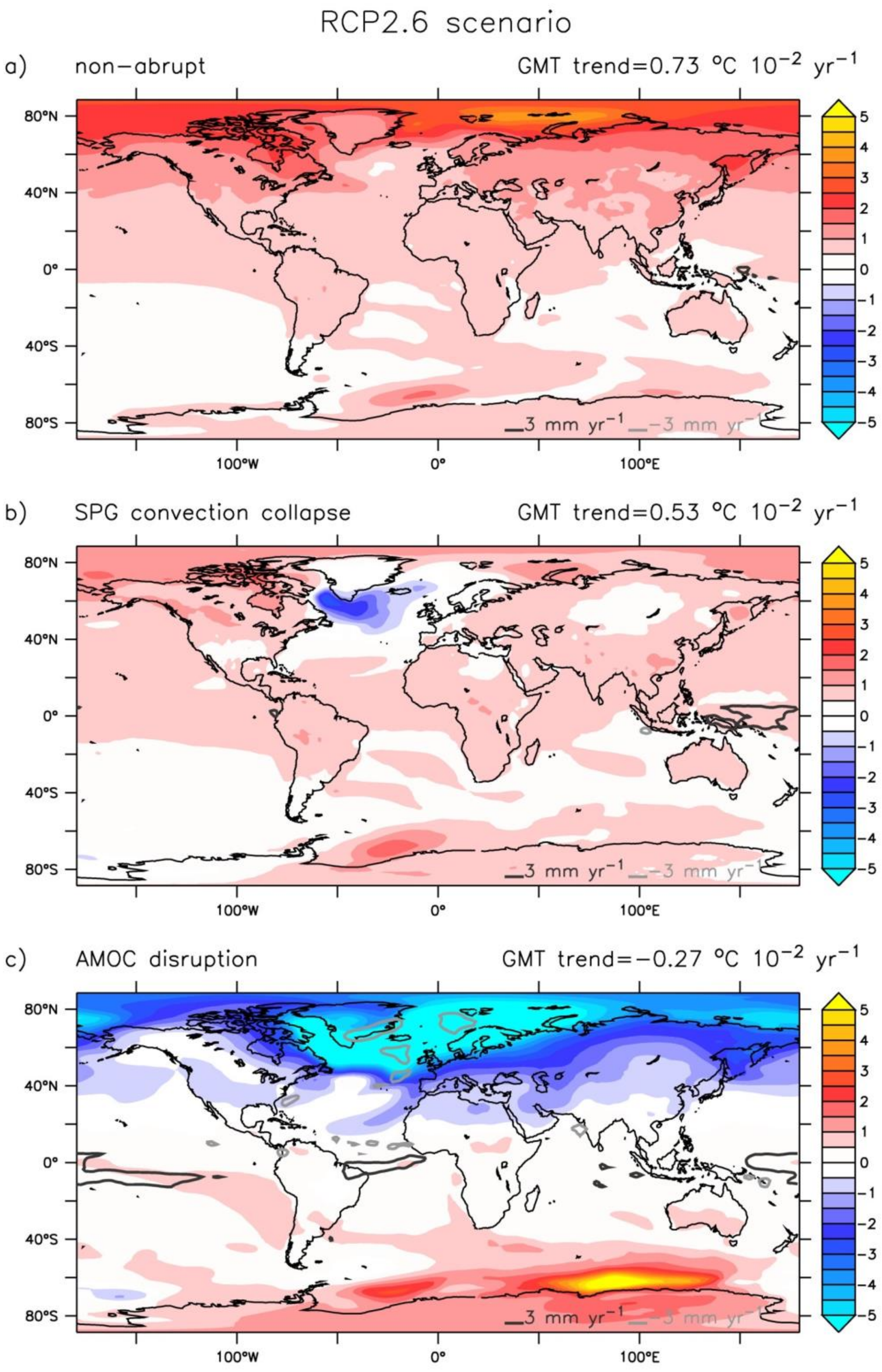


**Supplementary Figure 7. Different climatic impacts in RCP2.6 scenario.** Same as for Fig. 4 but for RCP2.6.

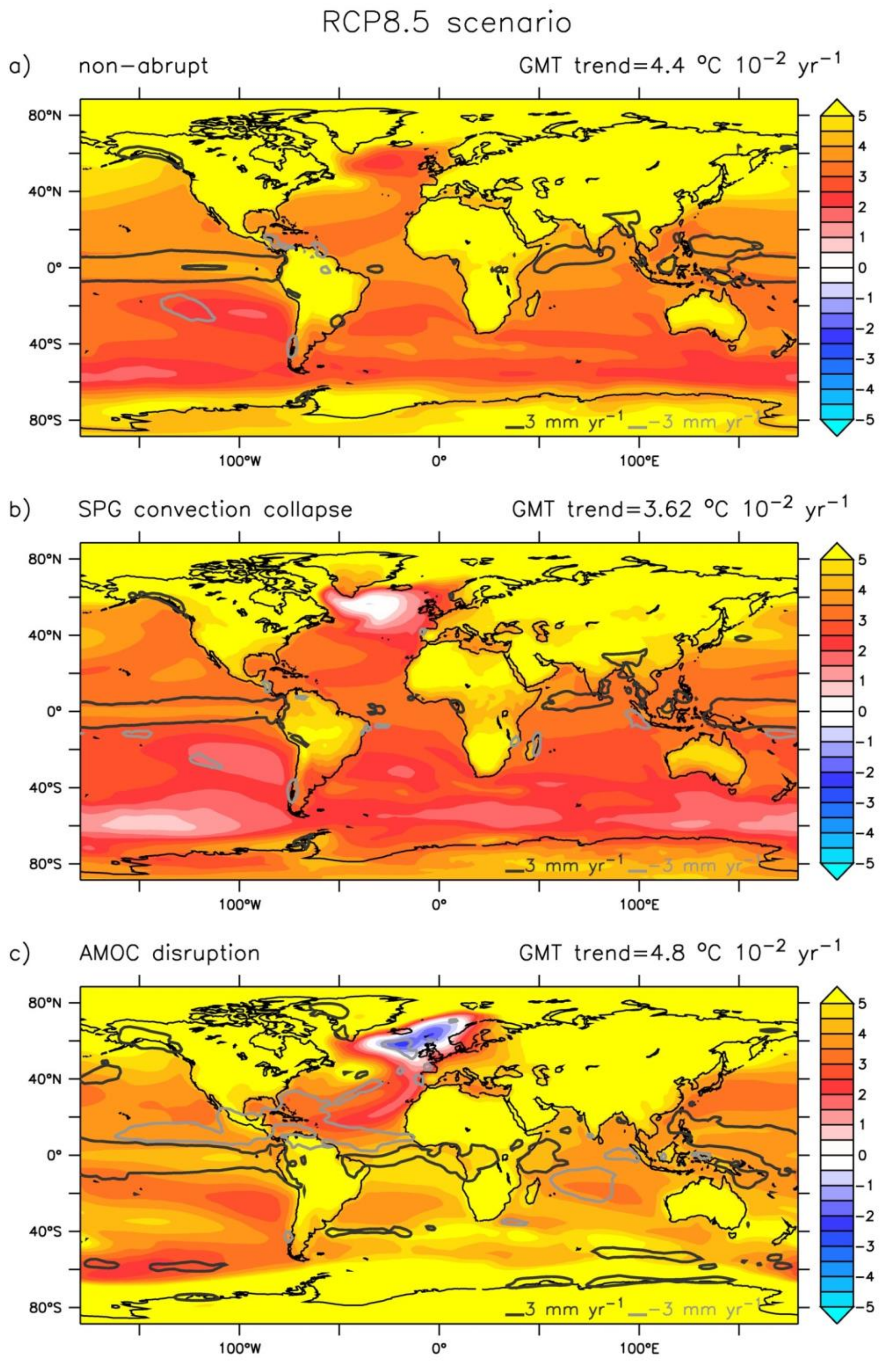


**Supplementary Fig. 8. Different climatic impacts in RCP8.5 scenario.** Same as for Fig. 4 but for RCP8.5.

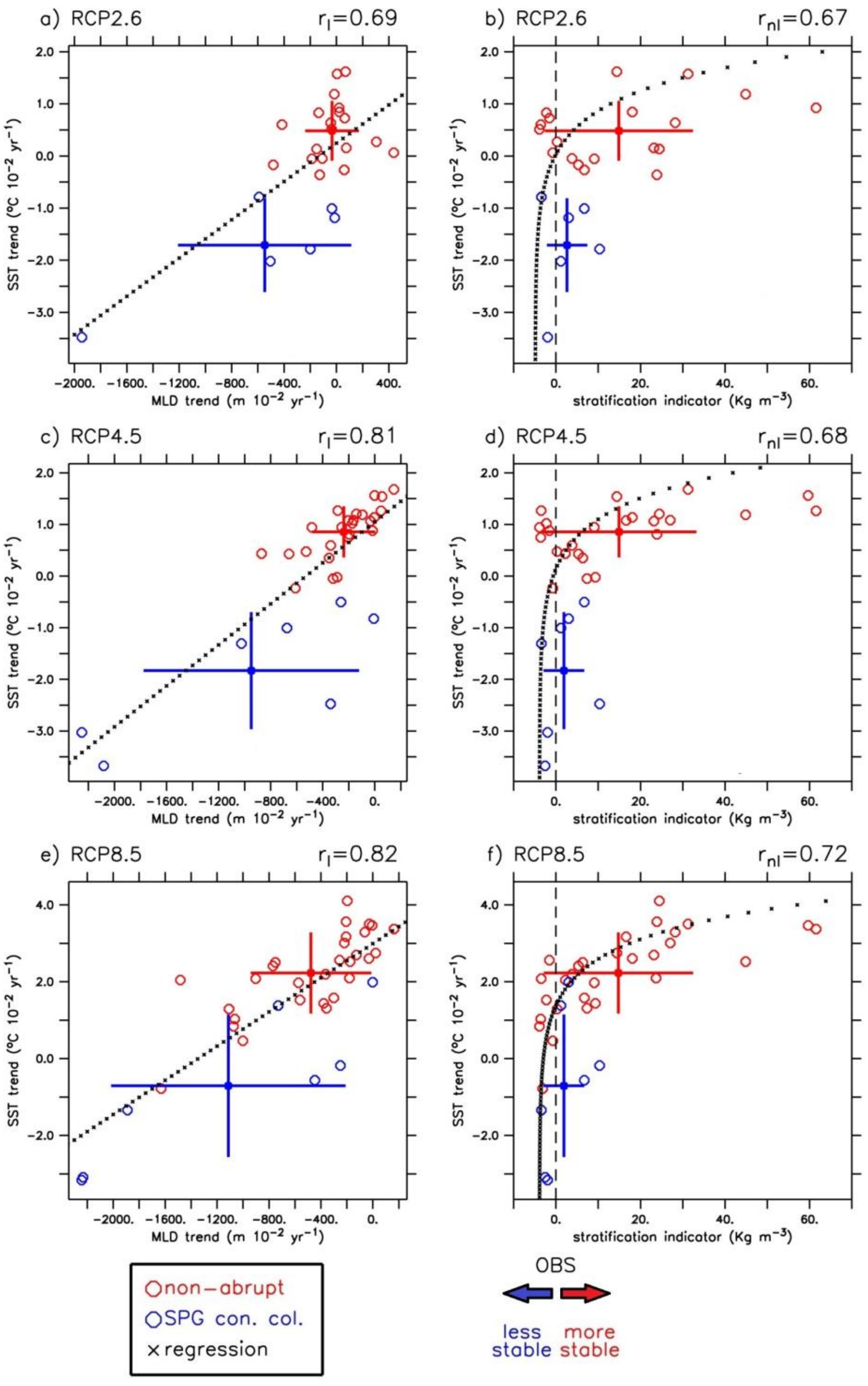


**Supplementary Figure 9. Use of an alternative reference region for the calculation of MLD and stratification index.** Same as Fig. 5 but with MLD and stratification index calculated in the region for which annual maximum MLD>1000 m according to GLORYS reanalysis data (mean value over the period 1993-2012).

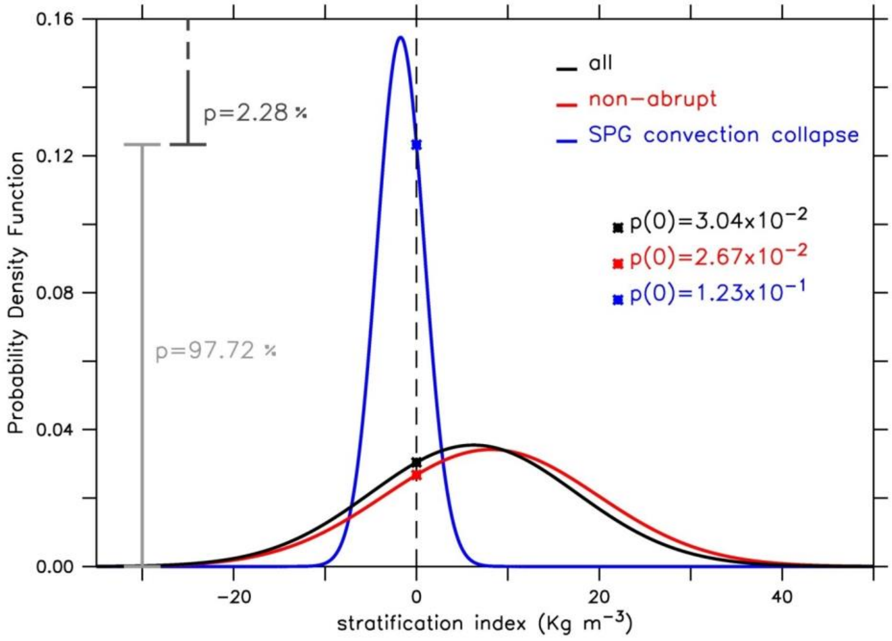


**Supplementary Figure 10. Significance of the differences between the background stratification in non-abrupt models and in SPG convection collapse models.** By assuming Gaussian distributions, curves represent probability density functions (PDF) for stratification indexes for non-abrupt sub-ensemble (red), for SPG convection collapse sub-ensemble (blue) and for all models ensemble (black). The intersection between PDF and dashed line at 0, *i.e.* stratification from observational data, indicates the likelihood that a specific subset of models is able to reproduce a SPG stratification matching that in observational data. Such a probability is higher for SPG convection collapse than for the non-abrupt models. However, this may be the consequence of the different sizes of the model ensembles, *i.e.* 7 members versus 29 members. To exclude sampling-flawed estimates we performed a statistical test based on Monte Carlo method. We standardized the sampling by choosing $10^4$ random combinations of 7 non-abrupt models among the 1560780 possible combinations, *i.e.* $C_{7,29}$=29!/(7!*22!), and calculated their PDF. We repeated this procedure 100 times finally finding that only 2.28±0.19% of the combinations of 7 non-abrupt models produce higher PDF values for null stratification index than in the SPG convection collapse ensemble, as elucidated by the interval bars on the left side of the panel. This test evidences that SPG convection collapse models reproduce a SPG background stratification that is closer to observational than that simulated by non-abrupt models, with significance higher than 95%.

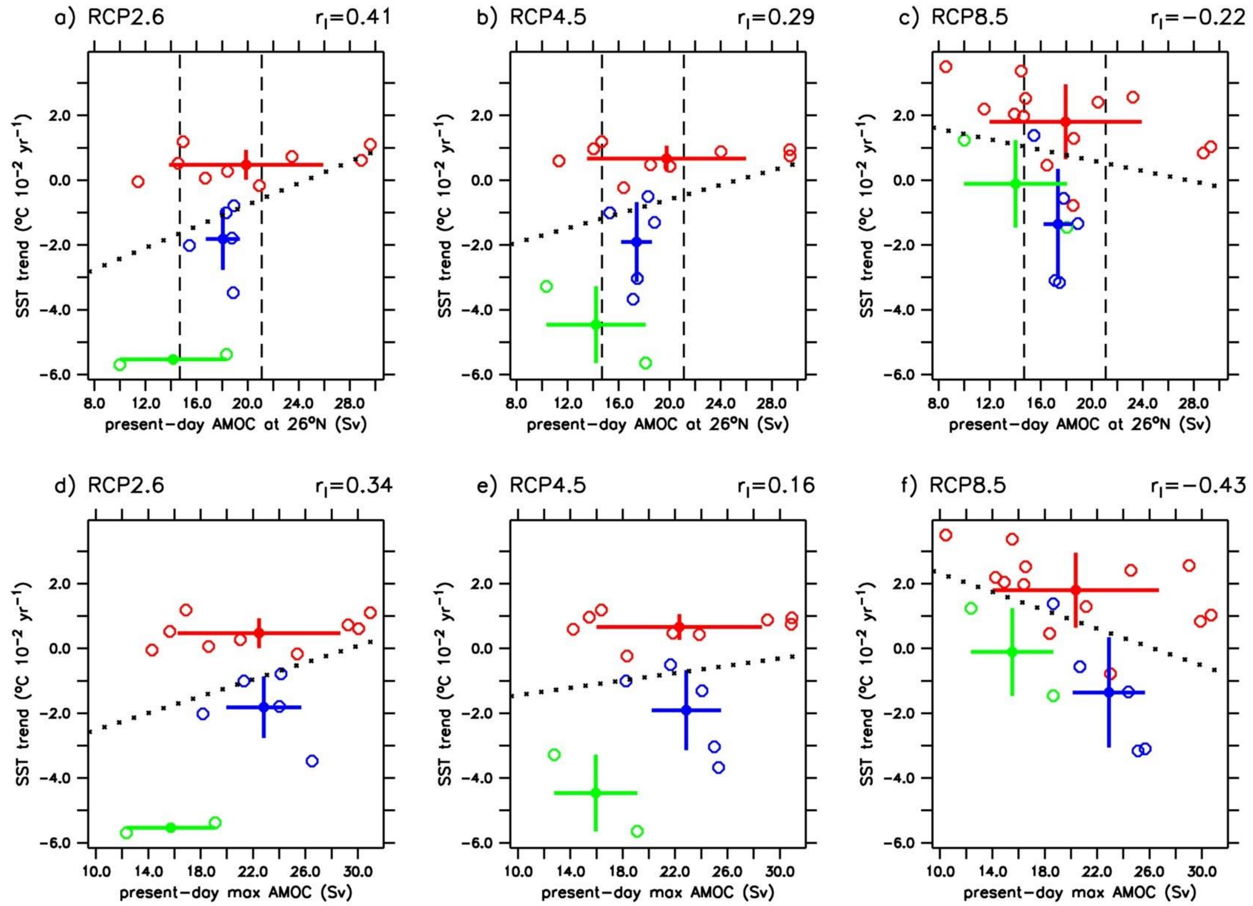


**Supplementary Figure 11. Evaluation of the present-day AMOC as an emerging constraint for the SPG SST response.** Scatterplot of the simulated SST trend (ºC 10$^{-2}$ year$^{-1}$) over the SPG under future RCP scenarios versus a) b) c) the simulated AMOC at 26ºN for present-day conditions (Sv) and d) e) f) maximum AMOC for present-day conditions (Sv). Dashed lines in a) b) c) delimit the range of the recently observed[8] AMOC at 26ºN, *i.e.* 17.9±3.2. Note that all the SPG convection collapse models lie within this range. The low statistical relations found evidence that the SST response over the SPG cannot be constrained by the present-day AMOC. This analysis is limited to those models for which AMOC data were directly available.

| Model | Skill Score S | SST trend (°C $10^{-2}$ $year^{-1}$) RCP2.6 | SST trend (°C $10^{-2}$ $year^{-1}$) RCP4.5 | SST trend (°C $10^{-2}$ $year^{-1}$) RCP8.5 |
|---|---|---|---|---|
| ACCESS1-0 | 0.86 | / | -0.02 | 1.44 |
| ACCESS1-3 | 0.89 | / | -0.05 | 1.31 |
| Bcc-csm1-1 | 0.37 | 0.84 | 1.14 | 2.61 |
| Bcc-csm1-1-m | 0.76 | 1.61 | 1.54 | 2.75 |
| BNU-ESM | 0.17 | 0.64 | / | 3.29 |
| CanESM2 | 0.00 | 1.19 | 1.19 | 2.52 |
| CCSM4 | 0.61 | / | 1.12 | 1.60 |
| CESM1-BGC | 0.63 | / | 0.97 | 1.06 |
| CESM1-CAM5 | 0.91 | -0.79 | -1.31 | -1.34 |
| CESM1-CAM5-1-FV2 | 0.92 | / | / | -0.78 |
| CMCC-CESM | 0.57 | / | / | 2.09 |
| CMCC-CM | 0.29 | / | 1.08 | 3.00 |
| CMCC-CMS | 0.67 | / | 1.08 | 3.17 |
| CNRM5-CM | 0.37 | -0.05 | 0.59 | 2.19 |
| CSIRO-Mk3-6-0 | 0.92 | -1.78 | -2.48 | -0.18 |
| EC-EARTH | 0.97 | / | 0.43 | 2.04 |
| FGOALS-g2 | 0.98 | 0.72 | 0.88 | 2.56 |
| FGOALS-s2 | 0.69 | -5.38 | -5.64 | -1.46 |
| FIO-ESM | 0.09 | -5.70 | -3.28 | 1.23 |
| GFDL-CM3 | 0.98 | -0.17 | 0.43 | 2.41 |
| GFDL-ESM2G | 0.99 | -1.19 | -0.83 | 1.98 |
| GFDL-ESM2M | 0.99 | -1.00 | -0.50 | -0.56 |
| GISS-E2-H | 0.85 | 0.83 | 1.02 | 1.53 |
| GISS-E2-H-CC | 0.87 | / | 1.26 | 2.08 |
| GISS-E2-R | 0.82 | -3.48 | -3.03 | -3.16 |
| GISS-E2-R-CC | 0.76 | / | -3.67 | -3.09 |
| HadGEM2-AO | 0.14 | -0.27 | / | 1.59 |
| HadGEM2-CC | 0.13 | / | 0.35 | 2.51 |
| HadGEM2-ES | 0.09 | -0.05 | 0.94 | 1.97 |
| IPSL-CM5A-LR | 0.11 | 1.57 | 1.68 | 3.50 |
| IPSL-CM5A-MR | 0.54 | 0.15 | 1.06 | 2.69 |
| IPSL-CM5B-LR | 0.00 | / | 1.56 | 3.46 |
| MIROC5 | 0.91 | -2.02 | -1.01 | 1.38 |
| MIROC-ESM | 0.12 | -0.36 | 0.81 | 3.56 |
| MIROC-ESM-CHEM | 0.08 | 0.13 | 1.20 | 4,01 |
| MPI-ESM-LR | 0.94 | 0.27 | 0.47 | 1.29 |
| MPI-ESM-MR | 0.96 | 0.06 | -0.23 | 0.46 |
| MRI-CGCM3 | 0.00 | 0.92 | 1.26 | 3.37 |
| NorESM1-M | 0.86 | 0.60 | 0.75 | 1.03 |
| NorESM1-ME | 0.84 | 0.51 | 0.94 | 0.84 |

**Supplementary Table 1. Skill scores and SST trends.** List of the skill scores of each model and corresponding SST trend over the SPG for the different scenarios. Models in red belong to the non-abrupt sub-ensemble, models in blue belong to the SPG convection collapse sub-ensemble and models in green belong to the AMOC disruption sub-ensemble.

**Supplementary Note 1:**

**Abrupt cooling in SPG convection collapse ensemble**

On the basis of the different SST responses in the SPG we defined 3 model sub-ensembles. Among them, SPG convection collapse models and AMOC disruption models project an abrupt cooling over the SPG. These rapid shifts involve changes in MLD over the convective regions and AMOC, which, along with the intensity of the SPG circulation and modes of atmospheric variability as the North Atlantic Oscillation (NAO), are strictly interconnected[1,2,3]. We summarize and detail here the main mechanisms underpinning the cooling events in SPG convection collapse models. According with our classification, this ensemble consists in 7 models, which, depending on their SST behaviour, can be further divided into 2 sub-groups, *i.e.* (*i*) those showing a single SST drop and (Supplementary Fig. 2), and (*ii*) those showing multiple SST drops (Supplementary Fig. 3).

On one hand, the SPG cooling events in CESM1-CAM5, GFDL-ESM2G, GISS-E2-R and GISS-E2-R-CC are characterized by a single temperature drop of around 2 $^{o}$C over 10 years (Supplementary Fig. 2a). These rapid changes take place in concurrence with a sudden decrease in sea surface density (Supplementary Fig. 2b). The latter is fully driven by a rapid freshening of the surface ocean (Supplementary Fig. 2c). The decrease in surface salinity reduces the MLD, leading to anomalies of *O*(1000 m), which obstruct the convective activity (Supplementary Fig. 2d). Moreover, by defining a SPG index as the local minimum of the barotropic streamfunction within the reference region, *i.e.* the maximum cyclonic transport within the SPG, we find that a transition of the SPG circulation to a weaker mode precedes the SST drop by a few years (Supplementary

Fig. 2f). A suspension of the SPG convection also affects the AMOC, which, however, does not dramatically decrease for these models (Supplementary Fig. 2e). A lead-lag analysis (not shown here) indicates that changes in surface salinity and MLD lead the rapid SPG cooling event rather than the AMOC.

On the other hand, CSIRO-Mk3-6-0, GFDL-ESM2M and MIROC5 feature strong high-frequency SST oscillations superimposed on a long-term weaker but persistent SST decrease (Supplementary Fig. 3a). These models are characterized by intermittent suspension of the convective activity, modulated by multiple oscillations of the MLD over a long-term decreasing trend (Supplementary Fig. 3d), which we suppose to be linked with similar responses of the SPG (although it can be shown only for CSIRO-Mk3-6-0 model due to lack of data for the others models.) This behaviour suggests a strong sensitivity to stochastic atmospheric conditions[4], e.g. the NAO.

Overall, we propose the following mechanisms driving a convection collapse-induced abrupt SST cooling in the SPG. The rise in radiative forcing yields a general increase of surface temperature and an enhancement of the hydrological cycle, which in the net precipitation area of the NA translates into a freshening trend (Supplementary Fig. 2c, 3c). Both warming and freshening contribute to a gradually decreasing sea surface density (Supplementary Fig. 2b, 3b), which, in turn, diminishes the mixed layer depth in the convective areas (Supplementary Fig. 2d, 3d). It is worth stressing that changes in Ekman-pumping due to a possible change in atmospheric winds are small (Supplementary Fig. 2i, 3i) and cannot explain the sudden change in mixed-layer depth. A thinner active layer in contact with the atmosphere decreases the heat capacity of the water column. As a consequence, the ocean cooling due to winter atmospheric

conditions becomes less effective at depth, leading a reduction in upward heat flux (Supplementary Fig. 2g, 3g). The oceanic heat loss is then confined to a shallower layer, resulting in a larger SST decrease during the winter that interferes with the background global warming trend. At the same time, less dense water at the core of the SPG reduces the density contrast with the surrounding lighter waters, thus slowing down the baroclinic cyclonic circulation (Supplementary Fig. 2f, 3f). A weaker mode of the SPG transports less subtropical salty water into the Labrador and Irminger Sea, further reducing the surface density there (Supplementary Fig. 2b, 3b). Moreover, a slower cyclonic circulation reduces the isopycnal outcropping in the centre of the SPG, which preconditions deep convection. Less convection in the SPG also causes the AMOC to slow down (Supplementary Fig. 2c, 3c), meaning a decrease in northward transport of subtropical salty and warm water masses (Supplementary Fig. 2h, 3h) into the NA. The interaction of these self-amplification processes of stratification pushes wide areas of the SPG across a threshold beyond which no deep convection is possible. The convection collapse therefore coincides with an SST drop, which locally overcompensates the warming contribution due to the rise in radiative forcing.

While this interplay of feedbacks with the stratification in the SPG applies to all SPG convection collapse models, the contribution of each single process to the local cooling is model dependent. Nevertheless, a common feature of these models is that a temperature drop over the SPG appears to be led by a sudden reduction of the local convective activity, but cannot be directly ascribed to a concurrent abrupt AMOC decline. The SPG convection and AMOC are strictly connected and therefore a reduction in AMOC plays an active role in these feedback mechanisms of SPG

stratification. Moreover, a collapse of the convective activity does affect itself the AMOC strength. However, in SPG convection collapse models the AMOC weakening (and the associated northward heat transport reduction) is comparable to that in models showing a continuous SPG warming trend (Supplementary Fig. 6c, 6d, 6e). This is because, after the SPG convection collapse, deep-water formation is still sustained (and even reinforced in some models) in other locations, i.e. Nordic Seas and/or Faroe-Shetland Channel (Supplementary Fig. 5). This explains, at least partially, why the AMOC does not strongly decrease in SPG convection collapse models, despite the interruption of deep-water formation in the Labrador/Irminger Sea. Thus, AMOC changes do not appear ultimately decisive in driving of the SST drop in the SPG convection collapse models. Rather, a rapid reduction of the upward heat flux from below (as a result of reduced convective mixing) appears to be the main cause of the abrupt SPG cooling in these models.

**Supplementary Note 2:**

**Abrupt cooling in AMOC disruption ensemble**

For FGOALS-s2 and FIO-ESM, cooling concerns a more extended area covering the whole northern NA. We identified such a cooling due to an effective reduction of the northward heat transport caused by an almost ceased AMOC. The AMOC strongly slows down already during the historical period (Supplementary Fig. 6b), and its decline lasts until the end of all the RCPs experiments. This produces the abrupt SPG cooling events evidenced in RCP2.6 and RCP4.5. For RCP8.5, the SST decline does not satisfy the criterion we used for defining an abrupt event. For this scenario indeed the competing effect due to the global warming is able to damp down the AMOC-induced

cooling[5].

An AMOC decline in response to global warming leads to a decrease in salinity and heat transport from the subtropical to subpolar NA. If the AMOC is a net salt importer during its route over the Atlantic basin, then a reduced northward salinity transport represents a positive feedback for the overturning circulation weakening itself. This self-amplifying process may potentially leads to extinction deep-convection activity in the NA[6] and a consequent AMOC disruption. However, FGOALS-s2 and FIO-ESM feature two clearly distinct convection sites. For FGOALS-s2 it is completely centred over the SPG and in the entrainment of the Greenland-Scotland overflows (Supplementary Fig. 5l, 5m) indicating that this model does not reproduce at all the convective activity over the Nordic Seas[7]. On the other hand, FIO-ESM features exactly the opposite configuration, with deep convection occurring exclusively in the Nordic Seas and an unrealistic absence of deep-water formation in the SPG (Supplementary Fig. 5n, 5o). Hence, both AMOC disruption models possess only one main site of deep convection, likely making the AMOC in these models overly sensitive to changes in local convective activity since is fed by such a unique sinking region. Thus, an initial decrease in the convection activity generates a local cooling and a progressive AMOC deceleration due to the positive salt advection feedback. This amplifies the cooling over the convection site, which, thereafter, intensifies and spreads gradually involving the whole northern NA. We can therefore infer that in FGOALS-s2 and FIO-ESM the abrupt events are mainly driven by self-amplification feedbacks involving the AMOC decline. However, the present-day AMOC strength simulated by these models significantly differs form the observed AMOC strength[8], *i.e.* the available data measured at 26$^{o}$N. Also, FGOALS-s2

and FIO-ESM do not plausibly simulate the NA convection, being both characterized by a not realistic single region of deep-water formation (Supplementary Figs. 5l-5o). These represent further evidences that an AMOC shutdown within the 21st century is very unlikely[9].

It is worth stressing that also in the non-abrupt ensemble some models feature only one site of deep convection. However, they do not project an AMOC disruption, thus evidencing that an unrealistic simulation of a unique site of convection is not a sufficient condition for an overturning collapse to occur under RCP scenarios. Furthermore, models in the SPG convection collapse always feature two (or more) deep convection sites. Overall it can be concluded that models featuring only one deep-convection site are more susceptible to an abrupt AMOC collapse than models featuring more than one deep-convection site. Also, models correctly reproducing two deep-convection sites never simulate an AMOC collapse under RCP scenarios, but they are the only models that could simulate an isolated SPG convection collapse.

**Supplementary References**

[1] Hakkinen S. & Rhines, P. B. Decline of the subpolar North Atlantic circulation during the 1990s. *Science*, **334**, 655-659 (2004).

[2] Gao, Y. & Yu, L. Subpolar gyre index and the North Atlantic Meridional Overturning Circulation in a coupled climate model. *Atmosph. and Oc. Sc. Lett.,* **1**, 29-32 (2008).

[3] Lohmann, K., Drange, H. & Bentsen, M. Response of the North Atlantic subpolar gyre to a persistent North Atlantic oscillation like forcing. *Clim. Dyn.*, **32**,

273-285 (2009).

[4] Born, A. & Mignot, J. Dynamics of decadal variability in the Atlantic subpolar gyre: a stochastically forced oscillator. *Clim Dyn*. **39**, 461-474 (2012).

[5] Drijfhout, S. Competition between global warming and an abrupt collapse of the AMOC in Earth's energy imbalance. *Nature Sci. Rep.* **5**, 14877 (2015).

[6] Stommel, H. Thermohaline convection with two stable regimes of flow. *Tellus*, **13**, 224-230 (1961).

[7] Lin, P. *et al.* An abrupt slowdown of Atlantic Meridional Overturning Circulation during 1915–1935 induced by solar forcing in a coupled GCM. *Clim. Past Discuss*., **10**, 2519-2546 (2014).

[8] Mielke, C., Frajka-Williams, E. & Baehr, J. Observed and simulated variability of the AMOC at 26$^{o}$N and 41$^{o}$N. *Geophys. Res. Lett*., **40**, 1159-1164 (2013).

[9] IPCC, 2013: Summary for Policymakers. In: *Climate Change 2013: The Physical Science Basis. Contribution of Working Group I to the Fifth Assessment Report of the Intergovernmental Panel on Climate Change* (2013).
